# Chitosan- and alginate-based biopolymeric composites for the adsorption of heavy metal ions from wastewater: modification strategies, composite architectures, adsorption behaviour and regeneration

**DOI:** 10.1039/d6ra08674h

**Published:** 2026-11-11

**Authors:** Moumin Aden, Abdirahman Elmi, Samaleh Idriss, Abdoulkader Ibrahim Idriss

**Affiliations:** a Research Centre, Faculty of Science, University of Djibouti Djibouti Republic of Djibouti mouminaden@hotmail.com; b Medicinal Research Institute, Djibouti Study and Research Center Djibouti Republic of Djibouti abdourahman.elmi@cerd.dj; c Rectorat de Besançon 10 Rue de La Convention 25000 Besançon France; d Department of Electrical and Energy Engineering, Faculty of Engineering, Université de Djibouti 1904 Djibouti

## Abstract

Chitosan and alginate offer complementary amino, hydroxyl and carboxylate functionality for the removal of dissolved metal contaminants. Their practical performance depends on chemical modification, network stability, solution composition and sorbent architecture. This narrative review examines modification strategies, composite architectures, adsorption behaviour and regeneration, with particular attention to chitosan–alginate hybrids. The review links polymer chemistry and fabrication to reported adsorption performance, distinguishing literature-reported capacities from evidence of mechanism and operational durability. A descriptive compilation identifies study-specific results and gaps in experimental reporting. It is not a normalised benchmark or a systematic meta-analysis. Hybridisation and functionalisation can improve selected properties, but the available cross-study data do not establish universal superiority over single-polymer sorbents or isolate donor chemistry from composition and test conditions. Langmuir and pseudo-second-order fits describe equilibrium and kinetic data without independently proving chemisorption or rate control. Within-study controls, explicit aqueous speciation, distinct desorption and capacity-retention metrics, and validation under competitive continuous-flow conditions are needed for defensible comparison and scale-up.

## Abstract 2

Chitosan–alginate composites show reported *q*_max_ values from 12 to 1331.6 mg g^−1^ across 34 studies. Capacity is set by grafted donor chemistry, not polysaccharide content, and pH selects among five removal mechanisms.

## Graphical abstract

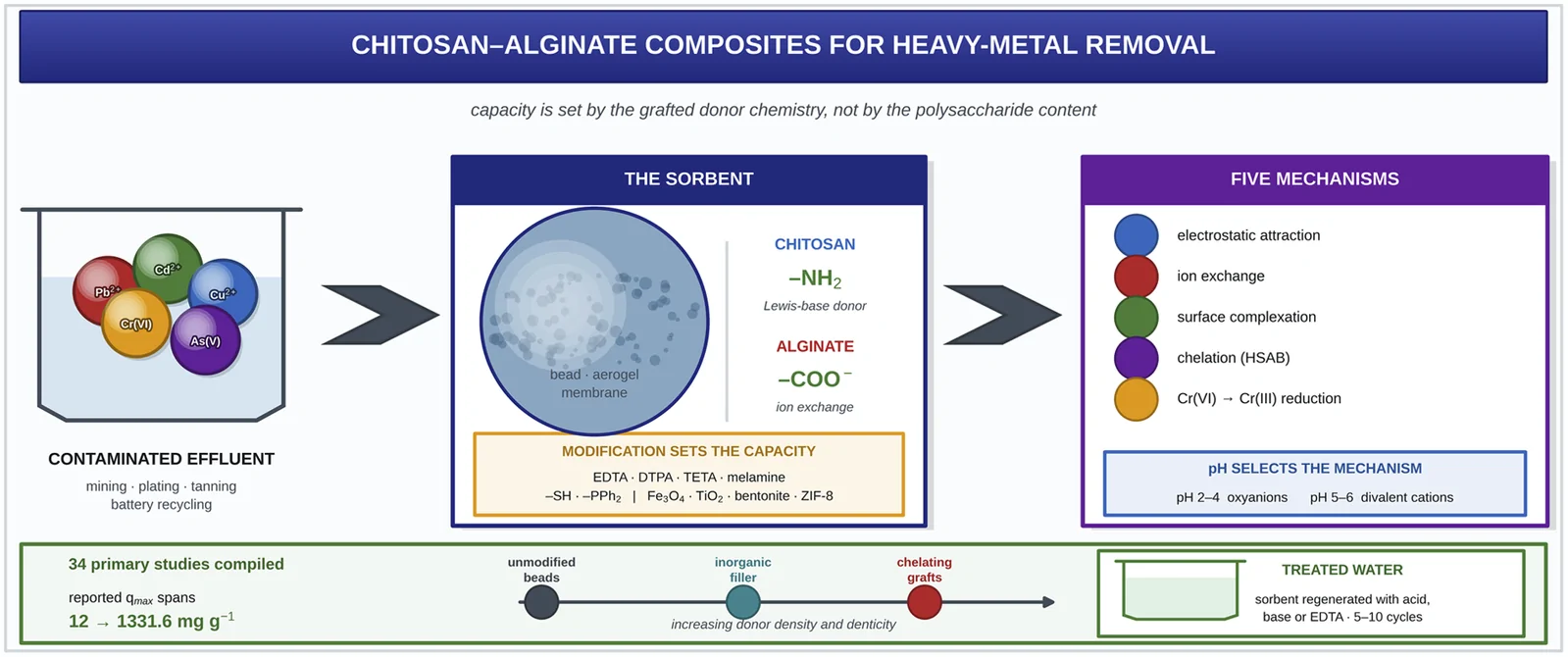

## 1. Introduction

Access to water of acceptable quality has become one of the defining constraints of the twenty-first century. Industrialisation, intensive agriculture and rapid urban growth discharge effluents loaded with organic and inorganic contaminants into receiving waters, and the inorganic fraction is dominated by heavy metal ions.^1,2^ Unlike most organic pollutants, metal ions are not biodegradable: they persist, they are transferred along trophic chains, and they accumulate in tissues, so that a discharge that appears dilute at the outfall can become a chronic exposure several trophic levels away.^3,4^

Chemical precipitation, membrane filtration, ion exchange, coagulation–flocculation, solvent extraction and electrochemical treatment each have application-dependent advantages and limitations.^2,4,5^ Adsorption can be useful for polishing dilute streams when affinity, mass transfer, regeneration and residual concentrations are compatible with the treatment target.^1,6^ No single treatment route is universally most economical; suitability depends on water composition, flow, discharge requirements and management of the concentrated waste stream.

The performance of an adsorption process is set almost entirely by the sorbent. Activated carbon remains the benchmark, but its production is energy-intensive, its regeneration is thermally demanding, and fine particles are difficult to recover from treated water.^7^ This has driven a search for sorbents built from renewable feedstocks, and polysaccharides have emerged as the most credible family. They are abundant, biodegradable, non-toxic, and—decisively—their polyhydroxylated backbones present functional groups that can be modified with a breadth of chemistry that few synthetic supports can match.^6,8,9^ Cellulose, starch, pectin, lignin, carrageenan, guar gum, chitin, chitosan and alginate have all been examined in this role.^5,10,11^

Within that family, chitosan and alginate occupy a special position, and for complementary reasons. Chitosan is a polycation: its glucosamine units carry primary amino groups that act as Lewis bases and form coordinate bonds with transition and post-transition metal cations, while the protonated form of the same group binds anionic metal oxyanions such as chromate and arsenate through electrostatic attraction.^12,13^ Alginate is a polyanion: its mannuronic and guluronic residues carry carboxylate groups that both bind divalent cations and provide the ionotropic gelation chemistry that turns a solution into a shaped, handleable bead in a single step.^14,15^ Combining them can form a polyelectrolyte complex that changes swelling, acid resistance and accessible binding sites; any improvement over the separate polymers depends on formulation and must be demonstrated using matched controls.^16,17^

Existing reviews address chitosan–alginate hydrogels,^16^ polymer modifications,^17,18^ cellulose-containing composites^19^ and composite beads.^20^ The contribution sought here is an evidence-focused account connecting fabrication and donor chemistry to three separate questions: what uptake was reported, what mechanism is independently supported, and what operational performance was demonstrated. Specifically, the review (i) relates fabrication route, network architecture and donor chemistry to the reported uptake (Sections 4 and 5); (ii) examines what isotherm and kinetic fits can and cannot establish, including linearisation, parameter identifiability and standard-state conventions (Section 6); (iii) separates single-metal uptake, percentage removal and regeneration metrics in a compilation that records the conditions missing from each entry (Section 1.1 and Tables 1–3); and (iv) sets out the evidence required to transfer batch results to fixed-bed, competitive and leaching-controlled operation (Section 6.5). The descriptive compilation is used to identify missing information and within-study contrasts, rather than to rank materials by capacities measured under different conditions.

Foundational reviews established metal coordination by chitosan and its derivatives,^21,22^ the chemistry and limitations of polysaccharide adsorbents,^23^ and the role of alginate-rich brown-algal biomass in metal biosorption.^24^ The present evidence audit follows the need to distinguish measurement, modelling and mechanism emphasised by Tran and co-workers.^25^

Sections 2–4 establish terminology, polymer structure and modification chemistry. Section 5 examines composite families and the limits of comparative evidence. Section 6 distinguishes equilibrium and kinetic modelling from mechanistic and regeneration evidence. Section 7 prioritises research gaps and future directions; Section 8 presents the conclusions.

### 1.1. Literature search and selection

This article is a critical narrative review; it is not a systematic review or a meta-analysis, and the structured search described here does not imply exhaustive coverage of the literature. The primary literature was searched in Scopus and the Web of Science Core Collection between December 2025 and May 2026; the last search was run on 14 May 2026. The Scopus query was applied to titles, abstracts and keywords (TITLE-ABS-KEY) and the Web of Science query to the topic field (TS); both combined terms for chitosan and its derivatives, alginate, adsorption, metal or metalloid targets and aqueous media (full search strings in Appendix A, Table 6). No date or language filter was applied, so the search dates do not define a publication window. The reference lists of relevant publications were also examined to identify additional studies. General references on the polymers, adsorption mechanisms and equilibrium or kinetic models were used to frame the discussion and were kept separate from the experimental corpus.

Primary studies were included when they reported (i) a material containing chitosan, alginate or their derivatives; (ii) application to dissolved metal or metalloid contaminants; and (iii) experimental adsorption results in aqueous media. Publications dealing exclusively with dyes, antibiotics or biomedical applications, and processes without a relevant evaluation of adsorption, were excluded from the experimental corpus; studies addressing metals together with organic contaminants remained eligible for their metal results. Review articles were retained as contextual sources but were not counted among the primary studies of the quantitative compilation. The absence of some experimental parameters did not automatically lead to exclusion; missing information is flagged and has not been estimated.

The database searches returned 310 records from Scopus and 250 from Web of Science (560 in total). After removal of 220 duplicates, 340 records were screened on title and abstract, of which 250 were excluded. Of the 90 full texts sought, five could not be obtained; of the 85 assessed, 61 were excluded on the criteria above, leaving 24 studies identified through the databases. Examination of reference lists identified 14 further publications, of which 10 were retained, giving a final corpus of 34 primary studies (Appendix A, Table 7). Screening and data extraction were carried out by the authors, and values were recorded as reported in the source articles.

The quantitative compilation (Tables 1–3) comprises 51 material–metal entries drawn from 34 primary studies published between 2016 and 2026; one publication contributes several entries when it reports several metals or formulations. Forty-eight entries from 32 studies concern chitosan–alginate hybrids. Three entries from two studies concern single-polymer architectures, a chitosan–Al_2_O_3_ hydrogel^56^ and two semi-interpenetrating networks based on alginate or on chitosan;^63^ they are retained as comparators and identified as such. For each entry the composition, target species, reported uptake, capacity or removal, study pH and reported isotherm model were recorded as given by the original authors, and values reported in molar units were converted to mass units. Because experimental conditions were not harmonised across studies, the compilation is used descriptively and not to rank materials (Section 5.3).

### 1.2. Terminology

The term heavy metals is used only as a conventional environmental descriptor, not as a chemically uniform class.^26^ The scope includes potentially toxic metal cations and metal or metalloid oxyanions, with oxidation state specified where known. Arsenic is a metalloid; Al^3+^ and rare-earth ions are discussed as additional target contaminants or recovery targets rather than classified by a density-based heavy-metal definition. Adsorption, overall removal, desorption efficiency and reuse capacity are treated as distinct quantities.

## 2. Adsorption of heavy metals from wastewater

Heavy metals reach surface and groundwater from mining and ore processing, metal finishing and electroplating, battery manufacture and recycling, tanning, pigment and dye production, fertiliser and pesticide application, and the combustion of fossil fuels. The toxicological case for removing them is unambiguous. Lead is a developmental neurotoxicant for which no threshold of safe exposure has been identified in children.^27^ Cadmium accumulates in the renal cortex over a biological half-life measured in decades and is associated with tubular dysfunction and skeletal demineralisation.^28,29^ Hexavalent chromium is a genotoxic carcinogen whose reduction to Cr(iii) inside the cell generates reactive intermediates.^30^ Inorganic arsenic is causally linked to skin, bladder and lung cancer,^31^ and mercury—particularly as methylmercury—targets the developing central nervous system.^32^ Selenium presents the inverse problem of an essential element with a narrow margin between requirement and toxicity, the World Health Organization guideline sitting at 40 µg L^−1^.^33^

Treatment selection depends on speciation, concentration, competing solutes, flow and the required residual concentration.^2,4,34^ Adsorption offers a useful polishing option when accessible sites retain sufficient affinity at the operating concentration.^1,5^ Batch capacity alone does not establish performance at trace concentration or demonstrate superiority over membrane separation, precipitation or ion exchange. Fig. 1 summarises material-level advantages and limitations.

**Fig. 1:**
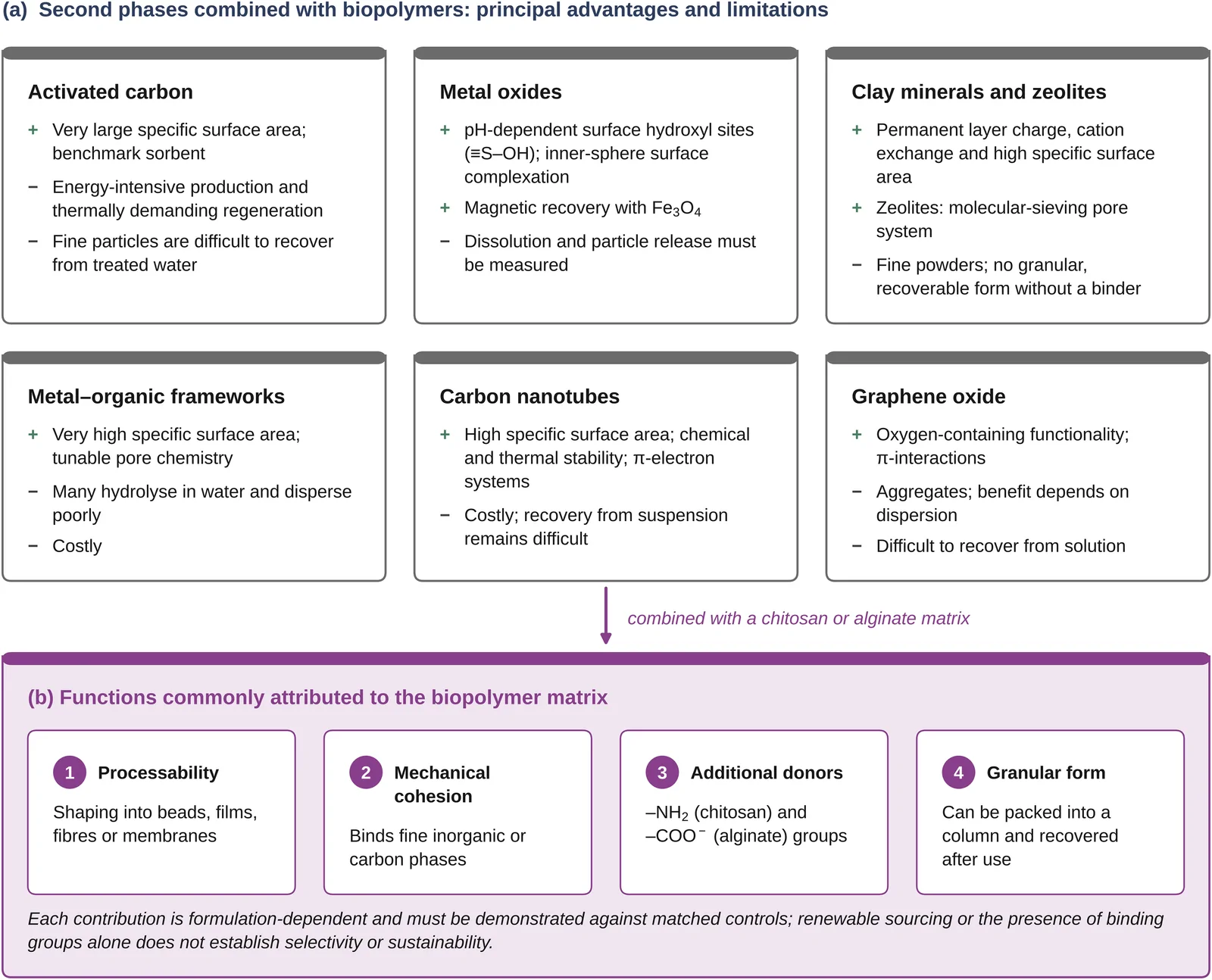
Adsorbent classes combined with biopolymers for heavy-metal removal. (a) Principal advantages and limitations of each second phase, as discussed in Sections 1, 2 and 5. (b) Functions commonly attributed to a chitosan or alginate matrix in such composites; each contribution is formulation-dependent and must be demonstrated against matched controls.

The sorbents deployed for this purpose have diversified considerably. Metal hexacyanoferrates remain the reference for caesium because their lattice cavity matches the hydrated radius of Cs^+^ almost exactly;^35^ zeolite-based nanocomposites exploit a combination of ion exchange and framework porosity;^36^ metal–organic frameworks offer specific surface areas and tunable pore chemistries that no natural material approaches, although their hydrolytic instability and poor dispersibility in water have proved a persistent obstacle;^37^ halloysite nanotubes provide a naturally tubular aluminosilicate with chemically distinct inner and outer surfaces;^38^ and hyper-cross-linked polymers provide permanent microporosity in an organic framework.^39^ Each of these has been combined with biopolymers, and in almost every case the biopolymer contributes what the inorganic phase lacks: processability, mechanical cohesion, additional chelating functionality, and a route to a granular form that can be packed into a column and recovered after use.

Applications outside dissolved-metal treatment are not evaluated here. The discussion focuses on adsorption and recovery of dissolved metal species, competitive uptake and the stability of the resulting sorbents.

Chitosan and alginate are useful chemical platforms for tuning affinity and material form. Neither renewable sourcing nor the presence of binding groups alone establishes selectivity, safety or process sustainability.

## 3. Chitosan and alginate: sources, structure and properties

Biopolymers are conventionally classified by chemical structure into polysaccharides, proteins and microbial polymers.^5,10^ The polysaccharide group—starch, chitin and chitosan, pectin, cellulose, alginate and the natural gums—is the one relevant to water treatment, because these materials are simultaneously abundant, non-toxic, biodegradable and chemically modifiable.^6,8,9^ Cellulose is the most abundant, chitin the second; alginate is distinguished by being extracted almost entirely from brown macroalgae, a biomass that is harvested at industrial scale and is not in competition with food production.^40,41^ The sources, extraction routes and molecular structures of chitosan and alginate are summarised in Fig. 2.

**Fig. 2:**
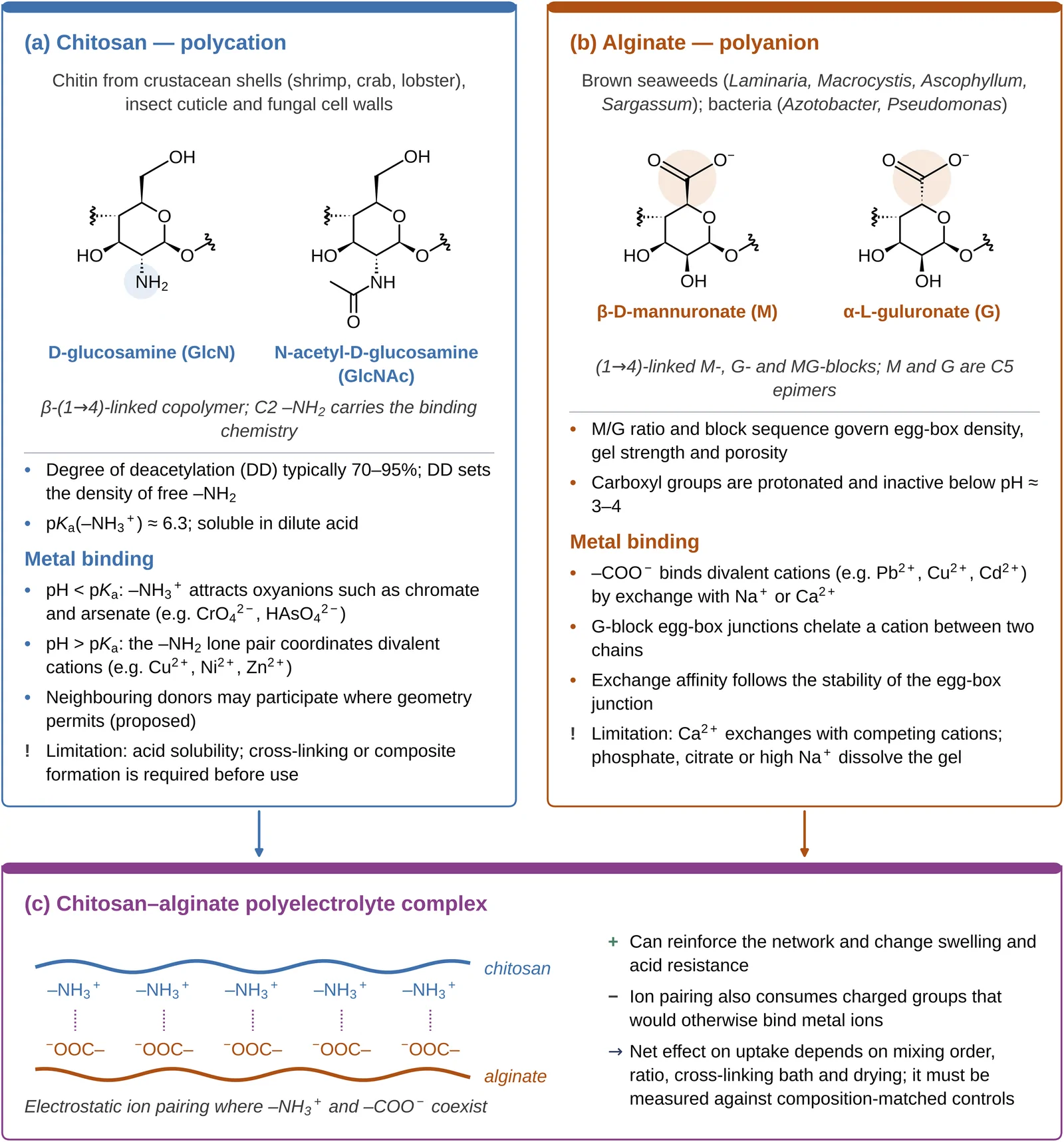
Origin, molecular architecture and metal-binding functionality of (a) chitosan and (b) alginate, and (c) the polyelectrolyte complex formed between them. Skeletal structures (wedge and hashed bonds give the stereochemistry; wavy bonds mark the continuing chain) show the substituent positions that carry the binding chemistry: the C2 amino group of glucosamine and the C6 carboxylate of the uronate residues, highlighted in the colour used for each polymer throughout. Binding modes marked as proposed are not demonstrated mechanisms.

For metal adsorption, the relevant structural variables are chitosan degree of deacetylation and molecular mass, alginate block composition, accessible functional groups, cross-link density, swelling and pore accessibility. Biomedical and packaging applications are outside the present scope.

### 3.1. Extraction and applications of biopolymers

#### 3.1.1. Extraction and applications of natural chitosan for adsorption of heavy metals

Chitin is recovered principally from crustacean processing waste—shrimp, crab and lobster shells—and secondarily from insect cuticle and fungal cell walls. The conventional route proceeds in four steps: demineralisation in dilute hydrochloric acid to dissolve calcium carbonate; deproteinisation in dilute alkali; decolourisation; and finally deacetylation in concentrated sodium hydroxide, which converts the *N*-acetyl-d-glucosamine units of chitin into the d-glucosamine units of chitosan.^13,42^ The typical sequence is shown in Fig. 3a. The conversion is deliberately incomplete: commercial chitosan is a copolymer, and its degree of deacetylation—usually between 70 and 95%—is the single parameter that governs the density of free amino groups and therefore the chelating capacity of the material.^12,13^

**Fig. 3:**
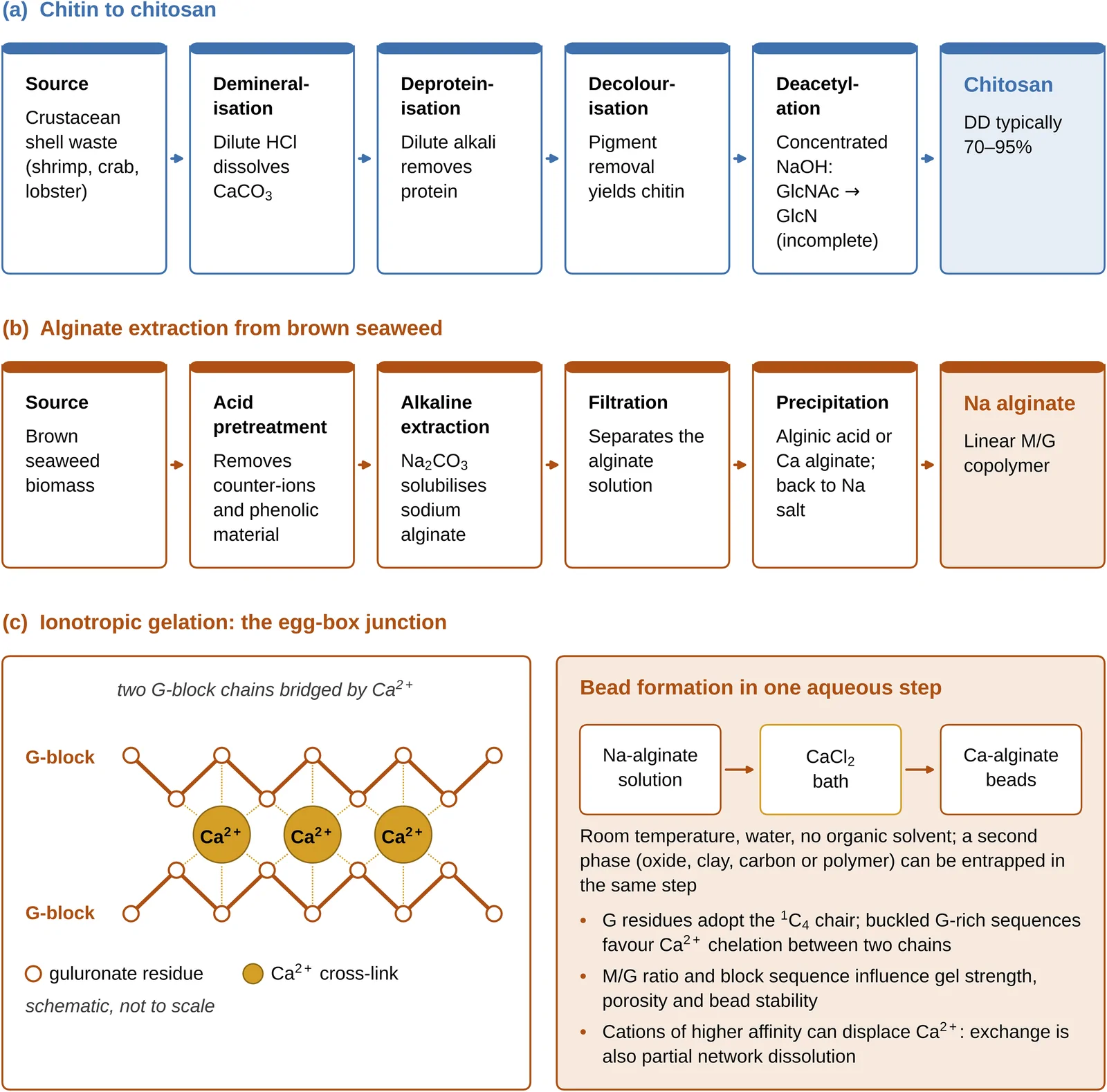
Preparation routes and gelation chemistry. (a) Recovery of chitin from crustacean shell waste and its deacetylation to chitosan; (b) extraction of sodium alginate from brown seaweed; (c) schematic egg-box junction between G-blocks and single-step formation of Ca-alginate beads. Only reagents named in Sections 3.1.1 and 3.1.2 are shown. G-rich sequences favour calcium-mediated junction formation; gel properties also depend on block sequence, cross-linking conditions and formulation.^45^

The consequence of this chemistry is a polymer whose behaviour changes character across the pH scale. Below the p*K*_a_ of the amino group, near 6.3, the chain is protonated, water-soluble in dilute acid, and electrostatically attractive towards anionic species; this is the regime in which chitosan sorbents remove Cr(vi) as chromate and As(v) as arsenate.^12,43^ Above the p*K*_a_ the free amine acts as an electron-pair donor and coordinates divalent cations directly. The same acid solubility that enables the first mechanism is, however, the principal practical weakness of native chitosan: beads disintegrate in the acidic effluents typical of electroplating and mining, which is why cross-linking or composite formation is effectively mandatory before use.^17,44^ A second limitation is selectivity. Unmodified chitosan discriminates poorly between metals of similar charge and radius, and shows little affinity for alkali and alkaline-earth ions, so its performance degrades in the high-ionic-strength matrices that characterise real industrial effluent.^8,18^

#### 3.1.2. Extraction and applications of alginate for adsorption of heavy metals

Alginate is extracted from brown seaweeds of the genera *Laminaria*, *Macrocystis*, *Ascophyllum* and *Sargassum*, and can also be produced bacterially by *Azotobacter* and *Pseudomonas* species.^14,40^ Industrial extraction proceeds by acid pretreatment to remove counterions and phenolic material, alkaline extraction with sodium carbonate to solubilise the polymer as sodium alginate, filtration, and precipitation as alginic acid or calcium alginate before reconversion to the sodium salt. The product is a linear copolymer of (1→4)-linked β-d-mannuronic acid (M) and α-l-guluronic acid (G) arranged in homopolymeric M-blocks and G-blocks and in alternating MG sequences.^15^

The block structure is not a detail but the controlling variable. G-rich sequences favour the buckled conformation that allows two chains to chelate a divalent cation between them in the so-called egg-box junction, so the M/G ratio determines gel strength, porosity and the stability of the resulting bead.^14,15,45^ This ionotropic gelation is what makes alginate uniquely convenient as a sorbent matrix: dropping a sodium alginate solution into a calcium chloride bath produces spherical, millimetre-scale, mechanically handleable beads at room temperature, in water, without organic solvent or thermal treatment, and the same operation can entrap a second phase—a metal oxide, a clay, a carbon or a second polymer—in a single step.^8,41^ Extraction and the egg-box mechanism are illustrated in Fig. 3b and c.

Against this, native alginate beads have real deficiencies. Their carboxylate groups are protonated and inactive below pH 3–4; the calcium cross-links are labile and exchange with competing cations or dissolve in the presence of phosphate, citrate or high sodium concentrations; and the wet gel has low mechanical strength and poor thermal stability, which limits column operation.^14,17^ As with chitosan, the practical answer has been modification and composite formation rather than use of the native polymer.

## 4. Modification of chitosan and alginate biopolymers

Modification can address acid solubility, gel instability or limited selectivity, with trade-offs in accessible sites, swelling and cost.^17,18^Fig. 4 provides the structure-to-performance framework. Cross-linking, grafting, functionalisation and format engineering are summarised in Fig. 5; their benefit requires a formulation-specific control.

**Fig. 4:**
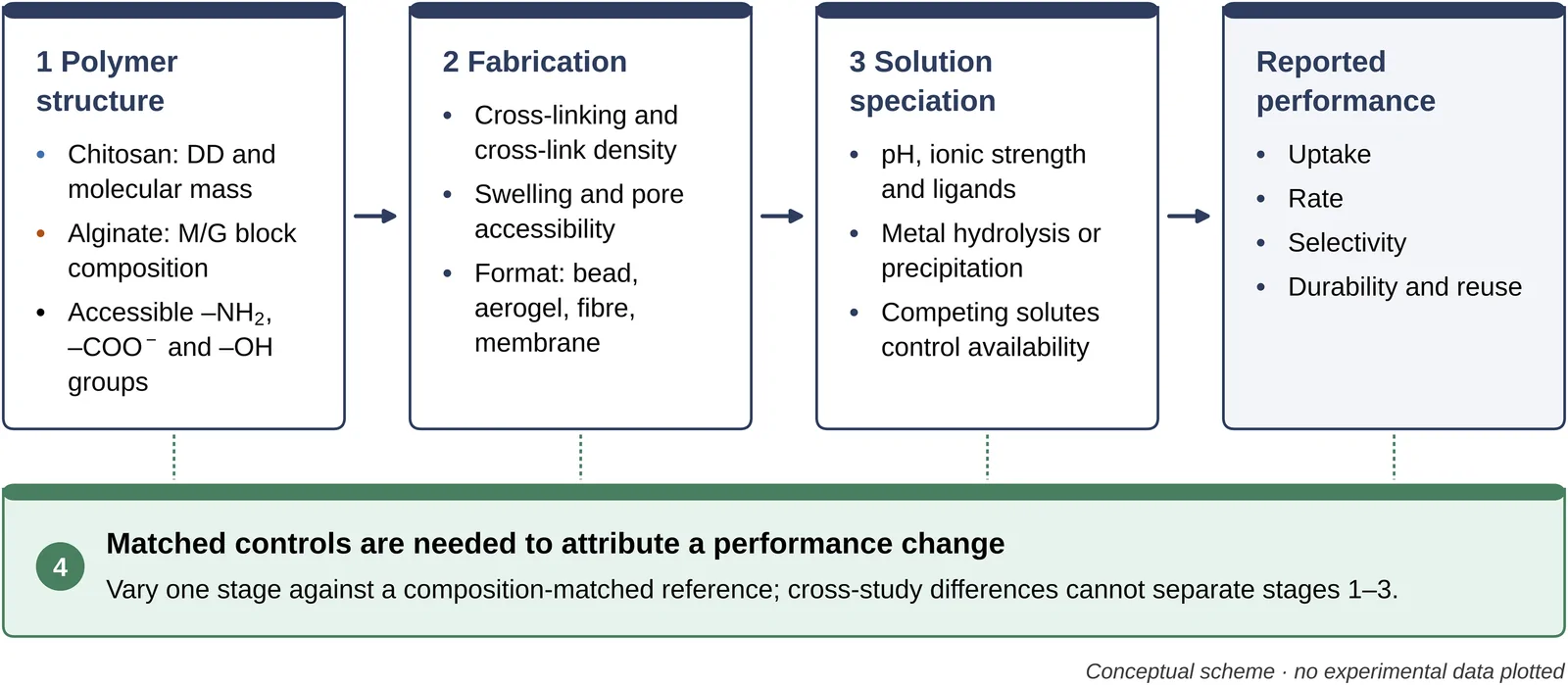
Conceptual sequence linking polymer structure, fabrication, solution speciation and the need for matched controls. This is an evidence framework rather than a causal model fitted to experimental data.

**Fig. 5:**
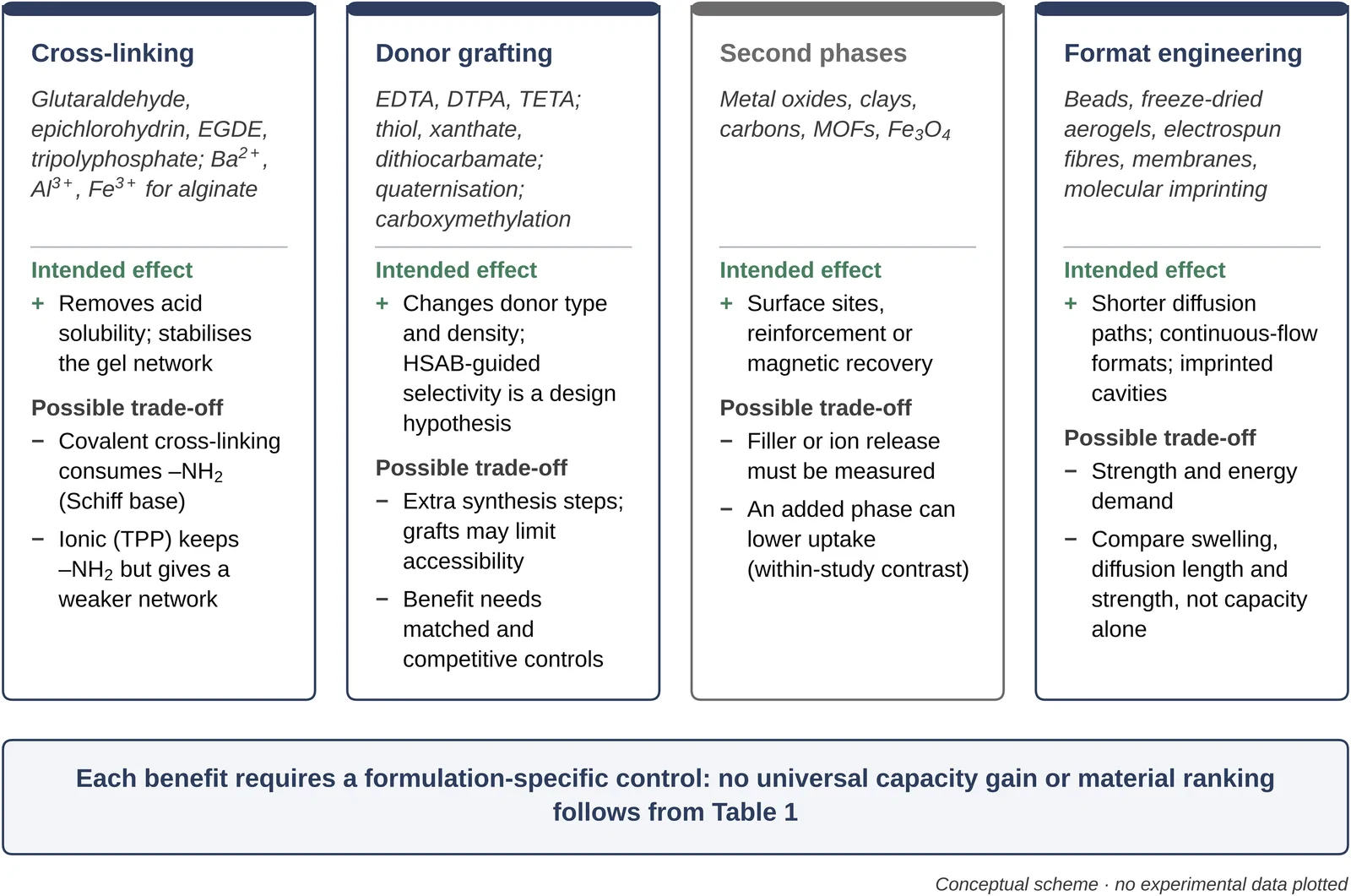
Modification strategies and their potential trade-offs in stability, accessibility, transport and containment. No universal capacity gain or material ranking is inferred. EGDE, ethylene glycol diglycidyl ether; TPP, tripolyphosphate; MOF, metal–organic framework; HSAB, hard–soft acid–base.

### 4.1. Modified chitosan biopolymer

Cross-linking is the first and most necessary modification, because it removes the acid solubility that otherwise disqualifies chitosan from use in the effluents where it is most needed. Glutaraldehyde, epichlorohydrin, ethylene glycol diglycidyl ether and tripolyphosphate are the standard reagents. Covalent cross-linking with glutaraldehyde consumes amino groups through Schiff base formation and therefore trades capacity for stability, whereas ionic cross-linking with tripolyphosphate preserves the amine but yields a less robust network.^17,46^ The choice between them is a genuine engineering trade-off rather than a matter of preference.

Grafting and functionalisation act in the opposite direction, adding donor atoms rather than consuming them. Aminopolycarboxylic chelators are the most productive class: ethylenediaminetetraacetic acid and diethylenetriaminepentaacetic acid introduce multidentate N,O-donor pockets that raise both capacity and selectivity, and their effect is quantified in Section 5.3. Polyamine grafts such as triethylenetetramine multiply the number of nitrogen donors per repeat unit; sulfur-bearing grafts—thiol, xanthate, dithiocarbamate—target soft acids such as Hg^2+^ and Cd^2+^, and the borderline acid Pb^2+^ in accordance with hard–soft acid–base reasoning; and quaternisation converts chitosan into a permanently cationic polymer whose affinity for chromate and arsenate no longer depends on solution pH.^12,17,18^ Carboxymethylation is a special case: it converts chitosan into an amphoteric polyampholyte bearing both amino and carboxyl groups, improving water solubility and broadening the working pH range, and carboxymethyl chitosan recurs throughout the composite literature for this reason.^12^

Two further routes deserve mention because they change the physical form rather than the chemistry. Polydopamine deposition provides a substrate-independent adhesive layer whose catechol groups are themselves strong metal chelators, and which serves as an anchoring platform for subsequent functionalisation.^47^ Electrospinning converts chitosan solutions into nanofibrous mats with specific surface areas far above those of beads and with correspondingly faster kinetics.^48^ Molecular imprinting, in which the target ion is present during cross-linking and subsequently eluted to leave a complementary cavity, is the most direct route to genuine selectivity in a multi-metal matrix,^49^ and membrane fabrication extends the same chemistry into a continuous-flow format.^50^

### 4.2. Modified alginate biopolymer

The modification logic for alginate differs from that for chitosan because the reactive handles differ: alginate offers carboxyl and hydroxyl groups but no nitrogen, so most strategies aim either to reinforce the gel network or to install nitrogen- or sulfur-based donors that the native polymer lacks.^17,18^

Reinforcement is achieved by substituting or supplementing the calcium cross-link. Higher-affinity cations—Ba^2+^, Al^3+^, Fe^3+^—produce more stable junctions than Ca^2+^; covalent cross-linking with glutaraldehyde or epichlorohydrin removes the reversibility of the ionic junction altogether; and interpenetrating or semi-interpenetrating networks formed with a second polymer confer mechanical properties that neither network achieves alone.^15,17^ Amidation of the carboxyl group with polyamines, and grafting of aminopolycarboxylic acids, install the nitrogen donors required for transition-metal chelation; thiolation serves the same purpose for soft cations.^18^ Graft copolymerisation with acrylic acid, acrylamide or acrylonitrile increases both the charge density and the swelling capacity of the network, and is the basis of most alginate superabsorbent sorbents.^51^

Chitosan–alginate complexation occurs over the pH interval where ammonium and carboxylate groups coexist. Mixing order, polymer ratio, concentration, cross-linking bath, contact time and drying history affect the resulting morphology and accessibility.^16,17,52^ Ionic pairing can reinforce a network while also consuming accessible charged groups. Consequently, the same preparation step may improve stability but reduce uptake, and its effect must be assessed against composition-matched controls. Bead extrusion, staged coating, freeze-drying and electrospinning should be compared through their effects on swelling, diffusion length, strength and energy demand, rather than capacity alone.

## 5. Biopolymeric composites for adsorption of heavy metals

A composite is formed when a biopolymer matrix is combined with a second phase that contributes properties the polymer does not possess. The motivation is consistent across the literature: dispersed fillers raise the specific surface area and introduce additional binding sites, they reinforce the network mechanically and thermally, and in the case of magnetic fillers they solve the separation problem that limits every fine-particle sorbent.^10,53–55^ The three families that recur are biopolymer/inorganic, biopolymer/polymer and biopolymer/carbonaceous composites, and this section follows that division for chitosan (5.1) and alginate (5.2) before treating chitosan–alginate hybrids separately (5.3). The characterisation strategy applied across these materials is summarised in Fig. 6.

**Fig. 6:**
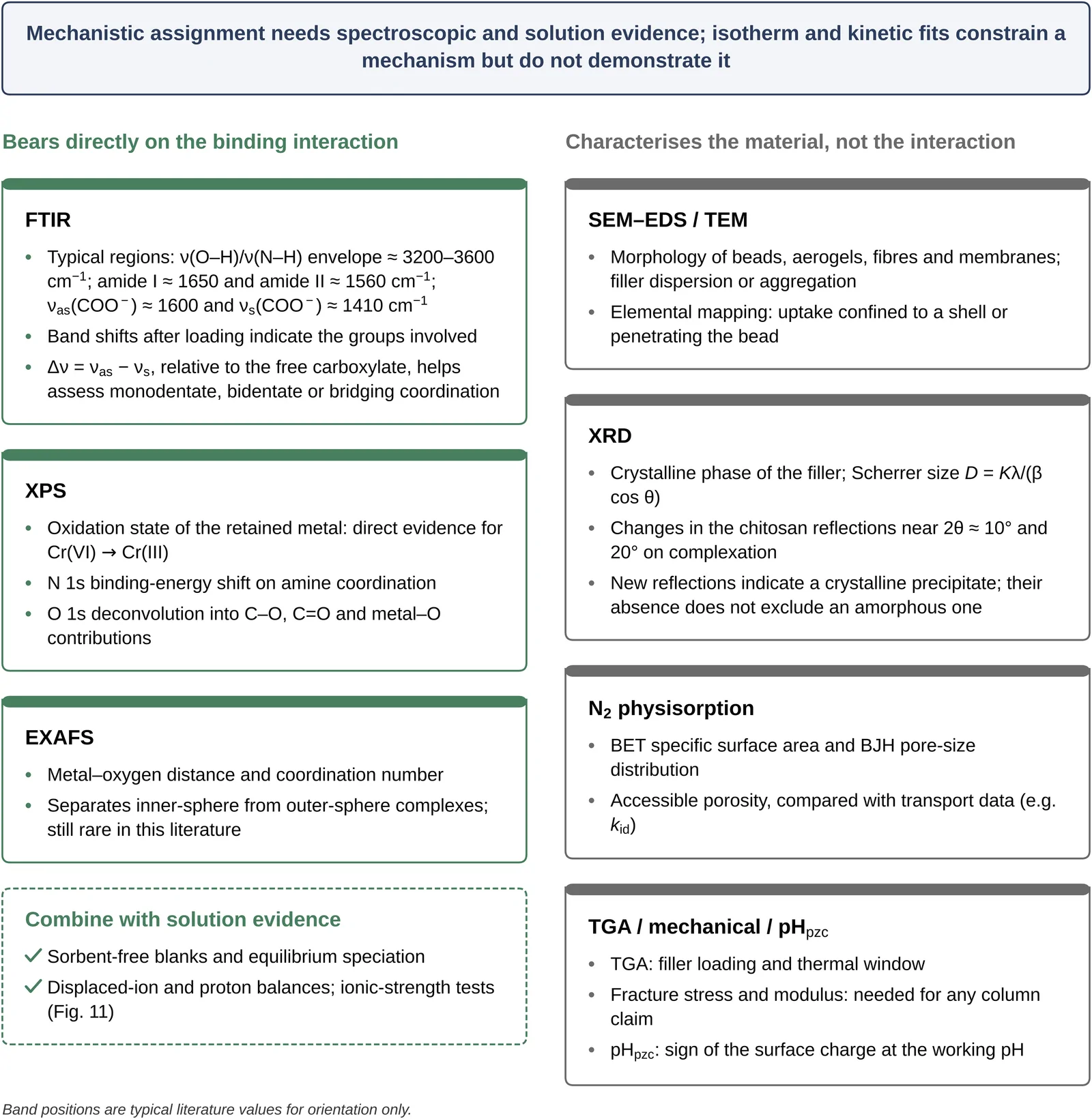
Characterisation strategy for chitosan- and alginate-based composites and the inference each technique supports. Fourier-transform infrared and X-ray photoelectron spectroscopies, complemented where available by extended X-ray absorption fine structure (EXAFS), bear directly on the binding mechanism; the remaining techniques characterise the material rather than the interaction. Band positions are typical values given for orientation only.

### 5.1. Chitosan biopolymeric composites for adsorption of heavy metals

Chitosan in its native state is not usable in practice: it is mechanically weak, it dissolves in acid, and it lacks selectivity for any particular hazardous metal in a complex matrix.^12,44^ Composite formation addresses all three deficiencies simultaneously, and the choice of the second phase determines which deficiency is addressed most strongly.

#### 5.1.1. Chitosan/inorganic materials composites

Metal oxides provide pH-dependent surface hydroxyl sites that can complement chitosan functionality. The chitosan–Al_2_O_3_ hydrogel study reports Cr(vi) uptake.^56^ Magnetic oxide and silica-containing architectures are reviewed elsewhere.^19^ A fitted isotherm does not independently identify the oxide-binding mechanism.

Aluminosilicates constitute the second major inorganic family. Bentonite and montmorillonite contribute a permanent negative layer charge, an interlayer accessible to hydrated cations, and a specific surface area that no biopolymer approaches; composites with lignin or polysaccharides have been reviewed specifically for water treatment,^57^ and the broader class of hybrid clay–biopolymer nanocomposites spans both environmental and biomedical applications.^58^ Halloysite offers the same aluminosilicate chemistry in a tubular morphology whose lumen and outer surface can be functionalised independently.^38^ Zeolite-based nanocomposites contribute a crystalline, molecular-sieving pore system.^36^ Magnetic chitosan materials, in which the inorganic phase is included principally to permit magnetic recovery, have been reviewed as a family in their own right.^59^

Metal–organic frameworks represent the most recent and most rapidly growing inorganic partner. Their appeal is obvious—specific surface areas and pore chemistries unattainable with natural materials—but their weakness is equally clear: many MOFs hydrolyse in water and disperse poorly, which is precisely what a biopolymer coating corrects. Chitosan and alginate stabilise the framework, improve processability, and add their own functional groups to the composite, and the resulting MOF–biopolymer materials have been reviewed as a distinct class.^37^ A concrete illustration in the hybrid system is given in Section 5.3, where sulfhydryl-modified chitosan is combined with ZIF-8 in an alginate matrix.^60^ Green routes to the inorganic phase have also been demonstrated: a chitosan/alginate nanocomposite incorporating phycosynthesised iron nanoparticles was applied to chromium remediation, the nanoparticles being generated biologically rather than by chemical reduction.^61^

#### 5.1.2. Chitosan/polymer composites

Polymer blending changes swelling and mechanical properties. Poly(vinyl alcohol)/chitosan magnetic composites are discussed in the secondary literature;^19^ without a directly checked primary study and a matched control, neither their numerical performance nor a causal effect of polymer blending is asserted here.

Acrylic monomers are the more productive route, because graft copolymerisation introduces carboxylate or amide functionality directly onto the chitosan backbone. Chitosan nanoparticle–alginate/methyl acrylate composite beads illustrate the approach in a hybrid system, giving 80.6 mg g^−1^ for Pb^2+^.^62^ Semi-interpenetrating networks built from poly(acrylic acid) and alginate, and from polyacrylamide and chitosan, have been compared directly within a single study and are discussed in Section 5.3.^63^ Reviews of polysaccharide-based hydrogels^16,64^ and of polysaccharide nanocomposite films^54^ collect the wider evidence for this family.

#### 5.1.3. Chitosan/carbonaceous materials composites

Carbonaceous fillers—activated carbon, biochar, graphene oxide and carbon nanotubes—contribute high specific surface area, chemical and thermal stability, and π-electron systems capable of interacting with both organic and inorganic solutes.^44^ Their combination with chitosan is doubly motivated: the carbon supplies surface area that the polymer lacks, and the polymer supplies the granular, recoverable form that fine carbon powders lack. This second point is the reason activated carbon is so frequently immobilised in chitosan or alginate; encapsulation lowers cost, improves mechanical properties and increases the number of functional groups relative to the carbon alone, and synergistic effects between polymer and carbon broaden the spectrum of adsorbable contaminants.^7^

Graphene oxide supplies oxygen-containing functionality and may change pore accessibility, but its benefit depends on dispersion and network interactions. Secondary reviews discuss graphene oxide/chitosan sorbents,^19,44^ while carbon-nanotube/polysaccharide composites are surveyed in ref. 65.

### 5.2. Alginate biopolymeric composites for adsorption of heavy metals

Sodium alginate has long been used as a starting point for metal sorption because of its hydroxyl and carboxyl density, and the composite route has been developed specifically to compensate for its low mechanical strength and limited thermal stability.^14,19^ The operational advantage of alginate over chitosan in composite fabrication is that ionotropic gelation entraps the second phase in a single, ambient-temperature step.

#### 5.2.1. Alginate/inorganic material composites

Clay–alginate materials combine ion exchange with an immobilising gel network. Bentonite, attapulgite and binary-metal uptake examples are reviewed in ref. 19 and 57. Competitive adsorption must be evaluated using component-resolved uptake and solution composition, rather than single-solute capacities.

Layered double hydroxides and magnetic fillers can alter surface chemistry and facilitate recovery.^66^ Their incorporation does not establish improved metal capacity or long-term stability. Magnetic carboxymethyl chitosan/alginate systems are included in the descriptive compilation.^67,68^ Selective recovery of critical materials is an adjacent application,^41^ but performance must be assessed against its own target-ion and matrix requirements.

#### 5.2.2. Alginate/polymer composites

Alginate/polymer blending can reinforce the network or introduce amine functionality. Polyaniline-containing systems may couple sorption with redox transformations, which require oxidation-state and mass-balance evidence.^19^ Polyvinylpyrrolidone-containing hybrids^69^ and polyethyleneimine-modified systems^70^ are discussed with the hybrid composites.

#### 5.2.3. Alginate/carbonaceous materials composites

Biochar, graphene oxide and other layered fillers can modify accessibility and mechanical behaviour in alginate composites.^7,19,65^ Claims of low cost or improved capacity require a common system boundary, verified primary data and controls.

### 5.3. Chitosan–alginate hybrid biopolymeric composites

Chitosan–alginate hybrids combine oppositely charged polymers and may integrate inorganic or grafted phases.^16,17,52^ Ionic pairing stabilises some formulations but also changes the number of accessible groups. Existing reviews provide context;^16,20^ their aggregated capacities and cycle counts are not treated here as evidence that hybrids consistently outperform the individual polymers. The rationale for hybridisation is property-specific rather than capacity-based: complexation with alginate is used to limit the acid solubility of chitosan, ionotropic gelation provides single-step shaping and entrapment of a second phase, and the combined network can change swelling, mechanical integrity and recoverability. Each of these properties requires its own composition-matched control, independently of the uptake capacity.

Tables 1–3 compile 51 material–metal entries from 34 primary studies (Section 1.1), including three single-polymer comparator entries.^56,63^ Entries are separated into single-metal uptake values (Table 1), percentage-removal values (Table 2) and an unresolved range across metals (Table 3), and the reported values are displayed in Fig. 7. The source studies do not consistently distinguish measured uptake from model-derived capacity, particularly for Freundlich fits, which have no finite saturation parameter; the values are therefore not suitable for quantitative benchmarking.

**Table 1:** Literature-reported single-metal uptake values and available conditions. Values labelled as capacity are not uniformly model-derived saturation capacities. This table is descriptive and is not a material ranking^a^

Biosorbent composite	Metal ion	Reported uptake or capacity/mg g^−1^	Study pH	Isotherm model	Ref.
Chitosan/calcium alginate/bentonite (CTS/CA/BT) hydrogel	Pb^2+^	434.89 mg g^−1^	5.0	Redlich–Peterson best fit; value reported as maximum capacity	71
Chitosan/calcium alginate/bentonite (CTS/CA/BT) hydrogel	Cu^2+^	115.30 mg g^−1^	5.0	Redlich–Peterson best fit; value reported as maximum capacity	71
Chitosan/calcium alginate/bentonite (CTS/CA/BT) hydrogel	Cd^2+^	102.38 mg g^−1^	5.0	Redlich–Peterson best fit; value reported as maximum capacity	71
Chitosan immobilised in alginate beads (ALG-CHz)	Cu^2+^	527.3 mg g^−1^^b^	5.0	Sips *q*_max_ (best fit); Langmuir 121.0	72
Chitosan immobilised in alginate beads (ALG-CHz)	Cd^2+^	207.0 mg g^−1^^b^	5.0	Sips *q*_max_ (best fit); Langmuir 113.9	72
Alginate–chitosan biopolymer composite (1 : 3 ratio)	Cd^2+^	≈212.9 mg g^−1^ (1.894 mmol g^−1^, exp.)	5.0 (initial)	Langmuir/Sips	73
Alginate–chitosan biopolymer composite (1 : 3 ratio)	Pb^2+^	≈218.4 mg g^−1^ (1.054 mmol g^−1^, exp.)	5.0 (initial)	Langmuir/Sips	73
EDTA-chitosan/alginate porous composite beads (EC-AB)	Pb^2+^	504.11 mg g^−1^ (exp.)	6	Langmuir (*q*_m,cal_ 492.97 mg g^−1^)	74
Chitosan microspheres/sodium alginate hybrid beads (CSM/SA)	Pb^2+^	189 mg g^−1^ (Langmuir; exp. 161)	5.2	Langmuir	75
Chitosan microspheres/sodium alginate hybrid beads (CSM/SA)	Cr(vi)	16 mg g^−1^ (Langmuir; exp. 14)	3.0	Langmuir	75
Chitosan/sodium alginate/nanocellulose (CSA-N)	Cd^2+^	≈384.4 mg g^−1^ (3419.5 µmol g^−1^)	n.s.	Freundlich	76
Chitosan/sodium alginate composite, no nanocellulose (CSA)	Cd^2+^	Up to ≈492.5 mg g^−1^ (4380.97 µmol g^−1^)	n.s.	Langmuir	76
Magnetic chitosan/sodium alginate gel bead (MCSB, Fe_3_O_4_)	Cu^2+^	124.53 mg g^−1^	5	Langmuir	77
Semi-IPN poly(acrylic acid)/alginate (PAA/alginate)	Cu^2+^	63.59 mg g^−1^	4.0	None fitted	63
Semi-IPN polyacrylamide/chitosan (PAAm/chitosan)	As(v)	17.8 mg g^−1^	5.0	None fitted	63
Chitosan–Al_2_O_3_ green hydrogel composite	Cr(vi)	48.9–51.5 mg g^−1^	2	Freundlich	56
Alginate/melamine/chitosan aerogel	Pb^2+^	1331.6 mg g^−1^	5.5	Langmuir	78
Multicavity TETA-chitosan/alginate composite beads (HCTA)	Cr(vi)	291.3 mg g^−1^	3.0	Freundlich best fit; exp. max	79
Chitosan–alginate–polyvinylpyrrolidone composite	Cr(vi)	208.67 mg g^−1^	4	Langmuir	69
Chitosan–alginate@Fe/Mn mixed oxide nanocomposite (Cs Alg@Fe/Mn)	Cr(vi)	455.93 mg g^−1^	4	Langmuir	80
Chitosan–alginate nanocomposite	Cr(vi)	39.59 mg g^−1^	5.0	Freundlich best fit; Langmuir *C*_max_ (linear)	81
Chitosan–alginate nanoparticles (CANPs)	Hg^2+^	217.4 mg g^−1^^b^	5	Langmuir	82
Mesoporous nanocellulose/sodium alginate/carboxymethyl-chitosan gel beads	Cu^2+^	169.94 mg g^−1^	n.s.	Langmuir	83
Mesoporous nanocellulose/sodium alginate/carboxymethyl-chitosan gel beads	Pb^2+^	472.59 mg g^−1^	n.s.	Langmuir	83
DTPA-chitosan/alginate composite beads (Ca-DTCS/ALG)	Cu^2+^	107.5 mg g^−1^ (exp.)	3.0	Sips best fit	84
TiO_2_@chitosan/alginate nanocomposite sponge	As(v)	202.27 mg g^−1^ (response-surface estimate)	5	Langmuir (parameters not in main text)	85
Chitosan–alginate + aspergillus australensis biomass beads	Cu^2+^	26.1 (exp.)–26.6 (Langmuir) mg g^−1^	5.0	Langmuir	86
Sodium alginate/sulfhydryl-modified chitosan@ZIF-8 gel beads	Cd^2+^	262.90 mg g^−1^	n.s.	Modified competitive Langmuir	60
Sodium alginate/sulfhydryl-modified chitosan@ZIF-8 gel beads	Ni^2+^	107.01 mg g^−1^	n.s.	Modified competitive Langmuir	60
Alginate/phosphine-functionalised chitosan beads (Alg/CSPPh_2_)	Cd^2+^	78.2 mg g^−1^	6.0	Freundlich best fit; exp. value	88
Alginate/phosphine-functionalised chitosan beads (Alg/CSPPh_2_)	Al^3+^	81.5 mg g^−1^	6.0	Freundlich best fit; exp. value	88
Chitosan/sodium alginate/Ca^2+^ double-network hydrogel (CTS/SA/Ca^2+^ PCDNH)	Pb^2+^	176.50 mg g^−1^	n.s.	Exp., single point; Freundlich best fit at 298 K	89
Chitosan/sodium alginate/Ca^2+^ double-network hydrogel (CTS/SA/Ca^2+^ PCDNH)	Cu^2+^	70.83 mg g^−1^	n.s	Exp., single point; Freundlich best fit at 298 K	89
Chitosan/sodium alginate/Ca^2+^ double-network hydrogel (CTS/SA/Ca^2+^ PCDNH)	Cd^2+^	81.25 mg g^−1^	n.s.	Exp., single point; Freundlich best fit at 298 K	89
Alginate–chitosan/hydroxyapatite from rock phosphate (Alg-Cs/HAP)	Cu^2+^	208.34 mg g^−1^	5	Langmuir	90
Magnetic bentonite/carboxymethyl chitosan/sodium alginate beads	Cu^2+^	56.79 mg g^−1^	5.0	Langmuir	67
Sodium alginate/carboxylated-chitosan/attapulgite aerogel-PEI (ATP/CCS/SA-PEI)	Cu^2+^	228.24 mg g^−1^	4	Langmuir best fit (*q*_m_ 302.89)	70
Chitosan nanoparticles/alginate/CMC/hydroxyapatite biocomposite	Cu^2+^	512.79 mg g^−1^^b^	6	Freundlich best fit; Langmuir *C*_ma*x*_ (linear)	91
Chitosan nanoparticles-alginate/methyl acrylate beads (CS NPs-Alg/MA)	Pb^2+^	80.6 mg g^−1^	5.3	Langmuir	62
Chitosan/calcium alginate/Fe_3_O_4_ hydrogel microsphere (CAF)	Cu^2+^	145.39 mg g^−1^	4.5–5	Langmuir	92
NaOH-modified chitosan/calcium alginate/Fe_3_O_4_ (NACAF)	Cu^2+^	261.31 mg g^−1^	4.5–5	Langmuir	92
Nanochitosan/sodium alginate/microcrystalline cellulose beads	Pb^2+^	114.47 mg g^−1^	6	Freundlich best fit; Langmuir *C*_max_	93
EDTA-modified chitooligosaccharide/sodium alginate/Ca^2+^ hydrogel (ECSDNH)	Zn^2+^	160.19 mg g^−1^	n.s.	Langmuir (298 K)	94
EDTA-modified chitooligosaccharide/sodium alginate/Ca^2+^ hydrogel (ECSDNH)	Ni^2+^	72.67 mg g^−1^	n.s.	Langmuir (298 K)	94
EDTA-modified chitooligosaccharide/sodium alginate/Ca^2+^ hydrogel (ECSDNH)	Mn^2+^	52.43 mg g^−1^	n.s.	Langmuir (298 K)	94

a n.s. = not specified in the source consulted, which does not necessarily mean unreported in the original article. Concentration interval, dose, temperature, ionic strength, contact time, wet or dry mass basis and capacity definition could not be recorded consistently and are not tabulated; their absence prevents normalisation. Values reported in molar units were converted with *M*(Cd) = 112.41 g mol^−1^ and *M*(Pb) = 207.2 g mol^−1^. Conditions for 31 of the 34 studies (all except ref. 60,69 and 76) were checked against the full texts and are given in Table S1 (SI); “exp.” denotes an experimental value and model-derived values are identified as such.

b Model-derived value exceeding the largest uptake attainable from the highest initial concentration and adsorbent dose used in the source (*C*_0,max_ V m^−1^; Table S1). For the other three studies, the conditions are those recorded from the sources consulted; where only the published abstract could be consulted, unreported conditions are marked n.s.

**Table 2:** Percentage-removal entries. These are not mass-normalised capacities^a^

Biosorbent composite	Metal ion	Reported metric	Study pH	Reported model	Ref.
Calcium alginate/carboxymethyl chitosan microbiocomposite (Ni_0.2_Zn_0.2_Fe_2.6_O_4_)	Nd^3+^	94.61% removal (predicted 95.72%)	5.5	Freundlich	95
Calcium alginate/carboxymethyl chitosan microbiocomposite (Ni_0.2_Zn_0.2_Fe_2.6_O_4_)	Tb^3+^	96.73% removal (predicted 96.17%)	5.5	Freundlich	95
Calcium alginate/carboxymethyl chitosan microbiocomposite (Ni_0.2_Zn_0.2_Fe_2.6_O_4_)	Dy^3+^	98.83% removal (predicted 99.44%)	5.5	Freundlich	95
Chitosan/sodium alginate biocomposite (CSAR3-L, freeze-dried)	Cu^2+^	94% removal (*q*_e_ 186.6 mg g^−1^); Langmuir *q*_ma*x*_ 300.3 mg g^−1^	6.0	Langmuir	96
Alginate/chitosan/glass bubbles adsorbent	Cu^2+^	≈80% (10 g in 500 mL, 20 min) and 87% (5 g in 500 mL, 125 min) removal at *C*_0_ = 4 mg L^−1^	n.r	None fitted	87

a n.s. = not specified in the source consulted, which does not necessarily mean unreported in the original article. Concentration interval, dose, temperature, ionic strength, contact time, wet or dry mass basis and capacity definition could not be recorded consistently and are not tabulated; their absence prevents normalisation. Values reported in molar units were converted with *M*(Cd) = 112.41 g mol^−1^ and *M*(Pb) = 207.2 g mol^−1^. Conditions for 31 of the 34 studies (all except ref. 60,69 and 76) were checked against the full texts and are given in Table S1 (SI); “exp.” denotes an experimental value and model-derived values are identified as such. For the other three studies, the conditions are those recorded from the sources consulted; where only the published abstract could be consulted, unreported conditions are marked n.s.

**Table 3:** Unresolved multi-metal uptake range. Component-specific values are not reported in the source consulted^a^

Biosorbent composite	Metal ion	Reported metric	Study pH	Reported model	Ref.
Magnetic alginate–bentonite/chitosan core@shell beads (mAB@Cs)	Pb^2+^, Cd^2+^, Cu^2+^, Zn^2+^, Ni^2+^, Hg^2+^, Co^2+^ (each tested separately)	12–20 mg g^−1^ (range across metals, *C*_0_ 50 mg L^−1^)	n.s.	n.s.	68

a n.s. = not specified in the source consulted, which does not necessarily mean unreported in the original article. Concentration interval, dose, temperature, ionic strength, contact time, wet or dry mass basis and capacity definition could not be recorded consistently and are not tabulated; their absence prevents normalisation. Values reported in molar units were converted with *M*(Cd) = 112.41 g mol^−1^ and *M*(Pb) = 207.2 g mol^−1^. Conditions for 31 of the 34 studies (all except ref. 60,69 and 76) were checked against the full texts and are given in Table S1 (SI); “exp.” denotes an experimental value and model-derived values are identified as such. For the other three studies, the conditions are those recorded from the sources consulted; where only the published abstract could be consulted, unreported conditions are marked n.s.

**Fig. 7:**
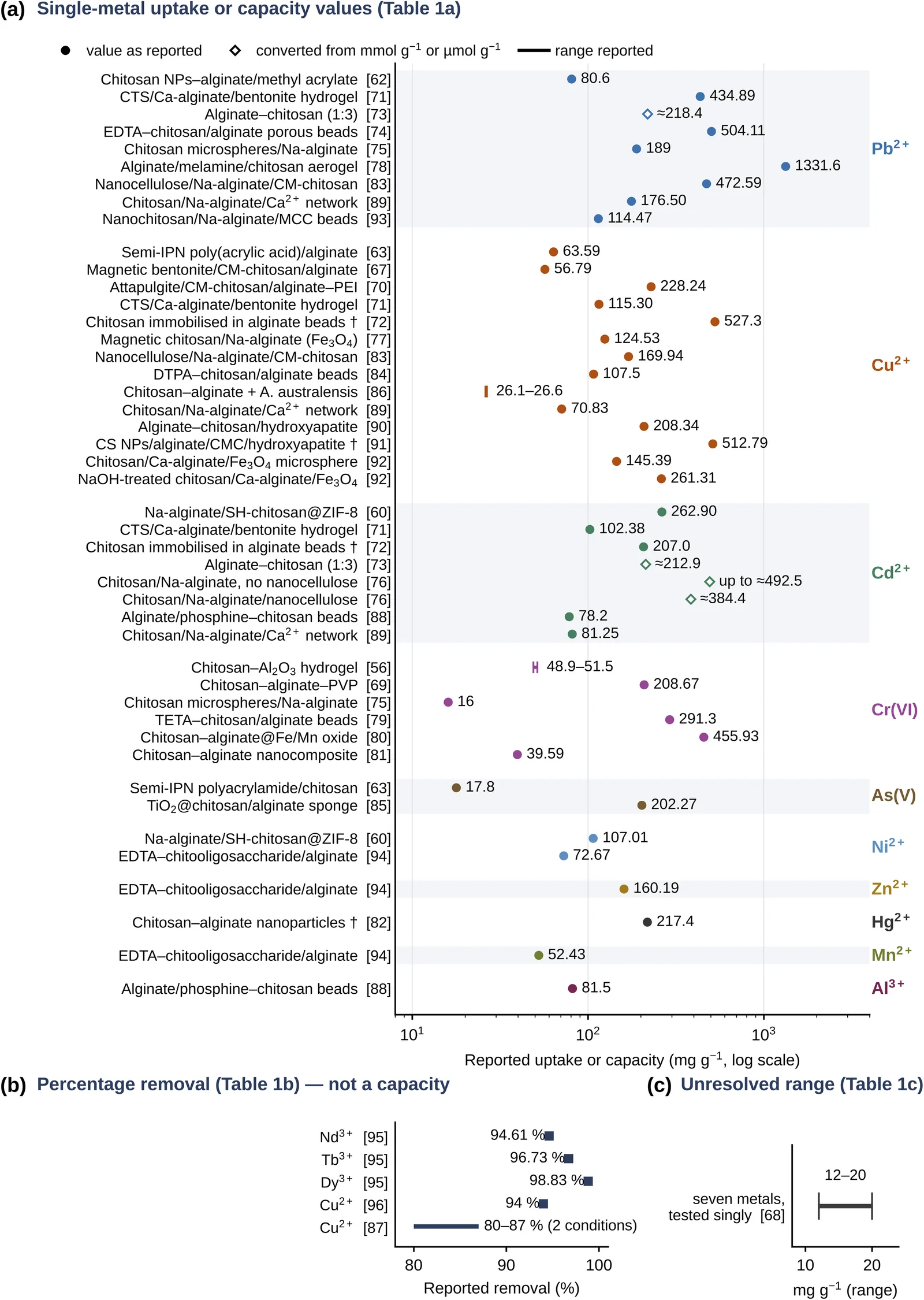
Descriptive inventory of the values compiled in Tables 1–3. (a) Single-metal uptake or capacity values (mg g^−1^, logarithmic axis), grouped by target ion and ordered by reference number; open diamonds denote values converted from molar units and horizontal bars denote ranges reported by the original authors. (b) Percentage-removal entries, which are not mass-normalised capacities. (c) Range across seven metals tested separately, without component-specific values in the text. All values were obtained under non-harmonised conditions (concentration range, dose, pH, temperature, mass basis); the figure does not rank the materials or demonstrate the intrinsic superiority of any of them. † Marks model-derived capacities that exceed the uptake attainable under the reported test conditions (ref. 72, 82 and 91).

The reported single-metal values span two orders of magnitude, from 16 to 1331.6 mg g^−1^ (Table 1 and Fig. 7a). This dispersion is descriptive. It combines differences in target species, formulation, measurement definition and experimental conditions; it cannot partition their causal contributions.

Chelating modifications are plausible routes to changing affinity and accessible donor density.^74,78,79,94^ However, the cross-study compilation cannot establish that donor chemistry dominates polysaccharide content, because composition and test conditions were not controlled across studies.

Inorganic phases may contribute surface sites, reinforcement or recoverability.^60,70,71,85,90^ Ref. 92 reports Cu^2+^ capacities of 145.39 and 261.31 mg g^−1^ before and after NaOH treatment of a chitosan/calcium alginate/Fe_3_O_4_ material. This within-study contrast concerns treatment of an oxide-containing formulation; it does not isolate the contribution of the oxide. Deprotonation, altered accessibility and a change in local pH are possible explanations. Hydroxide precipitation must be excluded before assigning the increase solely to adsorption.

Solution pH controls polymer protonation, metal hydrolysis and possible precipitation. No universal pH threshold separates adsorption from precipitation: the threshold depends on free-ion activity, total concentration, ligands, ionic strength and temperature.^97,98^ Cu^2+^ and Al^3+^ entries near pH 6 require particular scrutiny. In Tables 1 and 2 these are the Cu^2+^ entries reported at pH 5–6 (ref. 67, 72, 77, 90, 91 and 96) and the Al^3+^ entry at pH 6.0:^88^ at the millimolar concentrations typical of batch isotherms, these pH values can approach hydroxide saturation, so precipitation cannot be excluded from the information consulted. Appropriate checks include equilibrium speciation with stated constants and activities, sorbent-free blanks at the same pH, measurements of final pH and dissolved metal, and solid-phase characterisation. The extracted data do not include all the quantities needed for such a calculation, so no speciation result is claimed here; uptake reported for Cu^2+^ and Al^3+^ near pH 6 should not be attributed to adsorption alone until these checks are made.

Percentage removal is not a mass-normalised capacity. The rare-earth results in ref. 95 and the Cu removal results in ref. 96 and 87 are therefore separated from uptake values in Tables 2 and 3. Likewise, desorption efficiency is not capacity retention.

Application relevance depends on matching the feed matrix and operating regime, not on belonging to a particular capacity range. Studies involving simulated electroplating wastewater,^84^ simulated wastewater and an urban wastewater sample in which organic load, nutrients and bacteria, but not metals, were measured,^68^ competitive uptake^60^ and batch and column operation^95^ are useful starting points for this assessment. Their transferability requires verified concentrations, solution composition, hydraulics and breakthrough or cycling data. The chitosan–alginate beads containing *Aspergillus australensis* biomass were also subjected to a toxicological assessment,^86^ which matters for any drinking-water use, and a chitosan/alginate biocomposite containing CaCO_3_ was optimised through a designed experiment on its production factors.^96^

Within-study controls are more informative than unnormalised cross-study capacities. In ref. 76, about 492.5 mg g^−1^ is reported for Cd^2+^ without nanocellulose and about 384.4 mg g^−1^ with nanocellulose (converted from 4380.97 and 3419.5 µmol g^−1^). Because the two values derive from different isotherm models (Langmuir and Freundlich, respectively), they cannot quantify an effect of nanocellulose; they show only that the reported data provide no evidence that the added phase increased uptake. The distinct matched comparison in ref. 92 is discussed above without assigning a unique mechanism. Isotherm fit alone does not prove surface homogeneity, heterogeneity or chemisorption.

No universally best composite can be identified from Table 1. For selective recovery, compare competitive distribution coefficients and separation factors at a specified composition. For rapid treatment, compare loading at a common residence time and concentration. For packed beds, compare breakthrough capacity, treated bed volumes, pressure drop and regeneration cost. Chelator-bearing networks, magnetic beads and reinforced structures address different objectives; each requires matched controls and uncertainty estimates. Ref. 63 concerns different networks and different target ions and therefore cannot isolate a polymer effect through a direct capacity comparison.

For the families represented in Table 1, the evidence supports only qualitative distinctions. Networks carrying multidentate chelating grafts or sulfur- and phosphorus-donor grafts (EDTA, DTPA, TETA, melamine, sulfhydryl, phosphine)^60,74,78,79,84,88,94^ are designed for affinity and, where competitive data exist,^60^ for selectivity; their regeneration and long-term stability are not systematically reported in the compiled data. Composites with inorganic or magnetic phases (bentonite, attapulgite, hydroxyapatite, TiO_2_, Fe_3_O_4_, Fe/Mn oxides, ZIF-8)^60,67,68,70,71,77,80,85,90,92^ add surface-complexation sites, reinforcement or magnetic recovery, but require filler-release data (Section 6.5). Double-network hydrogels and porous aerogel or sponge formats^70,78,85,89,94^ target mechanical integrity and shorter diffusion paths, which should be documented by kinetic and mechanical data rather than by capacity alone. Because no study in Table 1 compares these families under identical conditions, the choice between them depends on the treatment objective, as outlined above.

## 6. Heavy metals removal: adsorption kinetics, isotherms, mechanism and regeneration

### 6.1. Equilibrium isotherms

An adsorption isotherm expresses the partitioning of the adsorbate between the solution and the solid at constant temperature, relating the residual equilibrium concentration *C*_e_ (mg L^−1^) to the specific loading *q*_e_ (mg g^−1^). Fitting an isotherm serves two purposes that are habitually conflated in this literature. The first is descriptive and unobjectionable: the fitted parameters interpolate the equilibrium relationship and supply the operating line needed to size a contactor. The second is inferential and requires far more care: the algebraic form of the successful model is read as a statement about the surface. That inference is weak, because the candidate models are not orthogonal—several are analytically nested, and all are smooth monotone functions capable of reproducing the same twenty or thirty experimental points within their scatter. A high coefficient of determination is therefore consistent with, but does not establish, the physical picture the model encodes.^99,100^ The functional forms discussed below are traced in Fig. 8, the criteria used to discriminate between them in Fig. 9, and the corresponding equations are collected in Table 4, which also lists the units of every parameter.

**Fig. 8:**
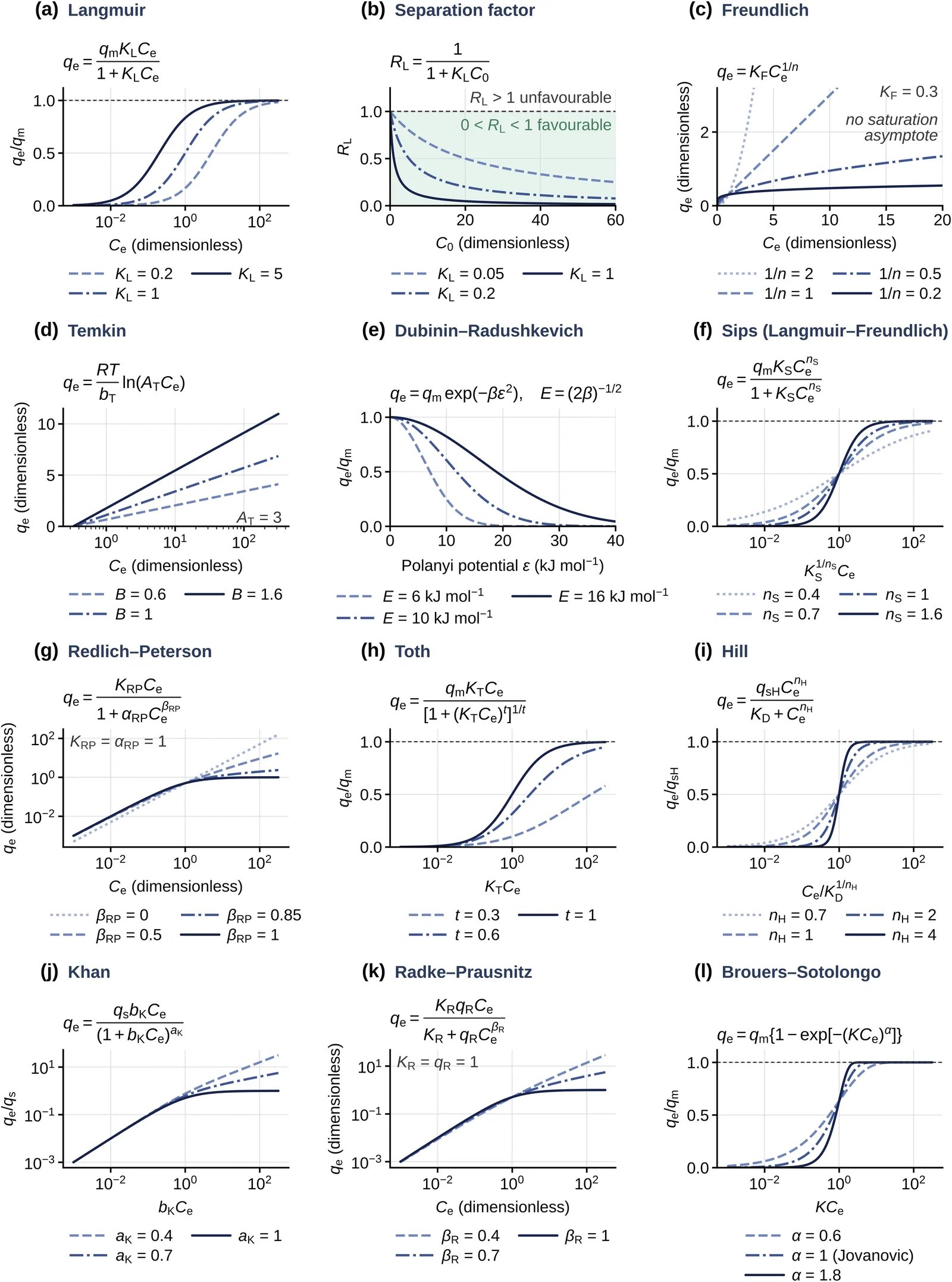
Equilibrium isotherm models applied to heavy-metal uptake on chitosan- and alginate-based composites: (a) Langmuir; (b) the Langmuir separation factor *R*_L_; (c) Freundlich; (d) Temkin; (e) Dubinin–Radushkevich; (f) Sips; (g) Redlich–Peterson; (h) Toth; (i) Hill; (j) Khan; (k) Radke–Prausnitz; (l) Brouers–Sotolongo and Jovanovic. The curves are the functional forms computed from the closed-form equations of Table 4 over dimensionless axes (panel (e) uses the Polanyi potential in kJ mol^−1^); the parameter values shown were chosen solely to make the shape of each model legible and are not experimental data.

**Fig. 9:**
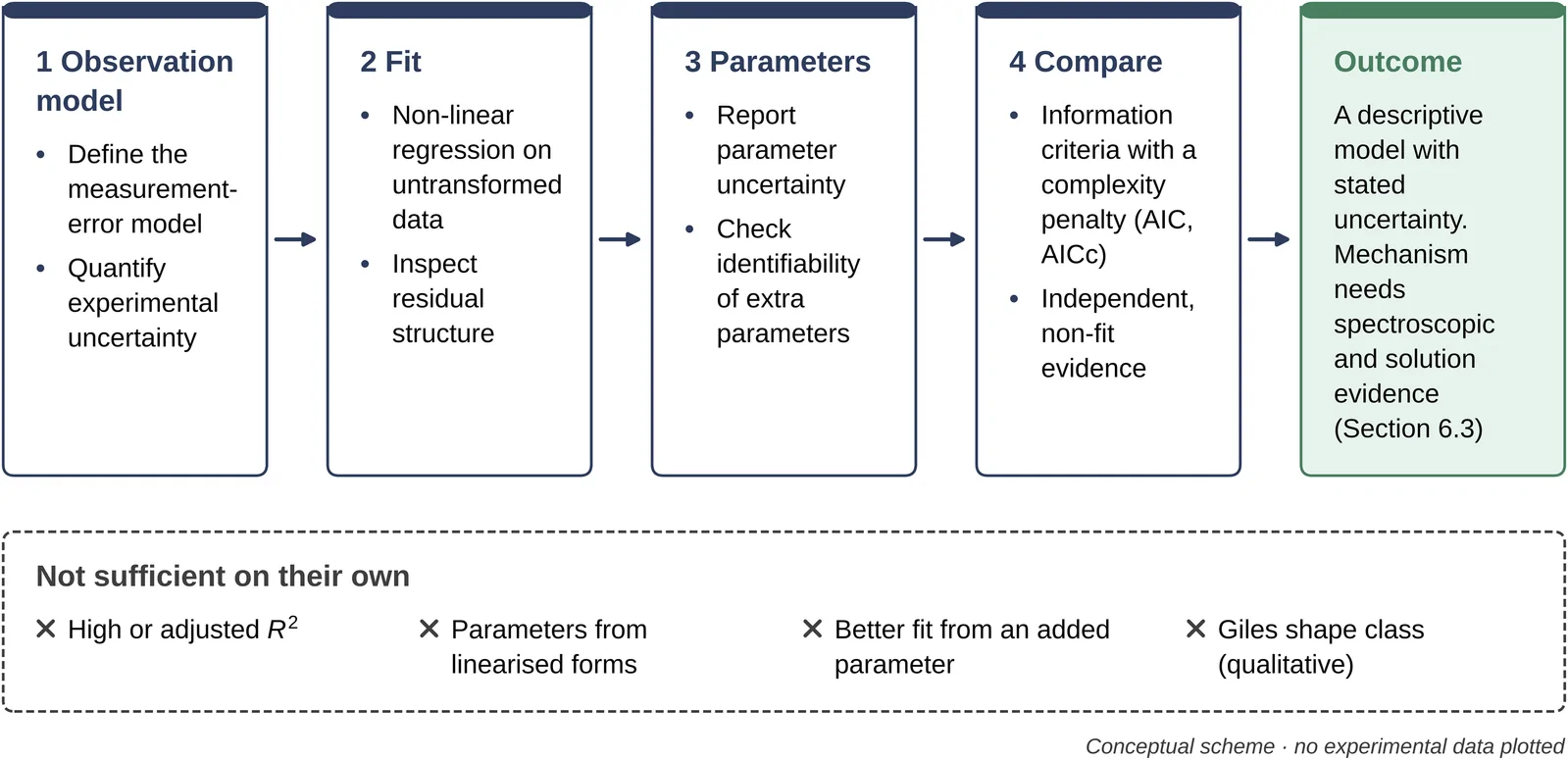
Model evaluation workflow. Fit an appropriate observation model, inspect residuals and parameter uncertainty, and compare complexity-adjusted criteria. A better fit does not independently identify adsorption mechanism.

**Table 4:** Equilibrium isotherm models used for heavy-metal uptake on biopolymeric composites: equation, adjustable parameters, the physical situation each model encodes, and its limiting behaviour^a^

Model	Equation	Parameters	Physical situation encoded	Limiting behaviour
Langmuir	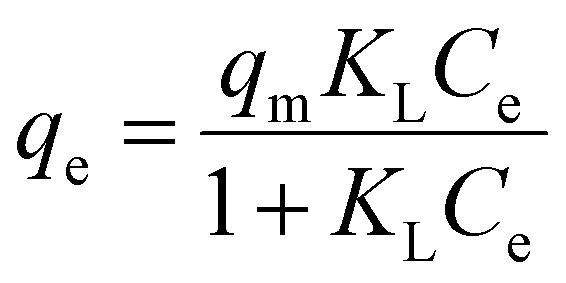	*q*_m_ (mg g^−1^)	Localised monolayer on energetically identical sites; no lateral interaction	Henry limit at *K*_L_*C*_e_ ≪ 1; plateau *q*_m_ at *K*_L_*C*_e_ ≫ 1
		*K*_L_ (L mg^−1^)		
Freundlich	*q*_e_ = *K*_F_*C*_e_^1/*n*^	*K*_F_ [(mg g^−1^)(L mg^−1^)^1/*n*^]	Empirical power law; no unique molecular interpretation	No saturation asymptote; 1/*n* → 0 approaches irreversible uptake
		*n* (—)		
Temkin	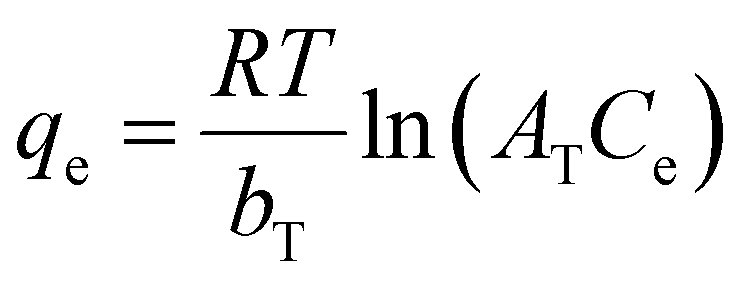	*A*_T_ (L mg^−1^)	Adsorption enthalpy falls linearly with coverage (adsorbate–adsorbate repulsion)	Undefined as *C*_e_ → 0; no plateau
		*b*_T_ (J *g* mol mg^−1^)		
Dubinin–Radushkevich	*q*_e_ = *q*_m_ exp(−*βε*^2^)	*q*_m_ (mg g^−1^)	Micropore filling over a Gaussian distribution of pore potentials	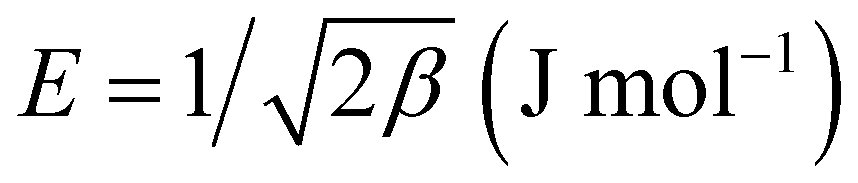 is model-dependent and does not classify mechanism independently
	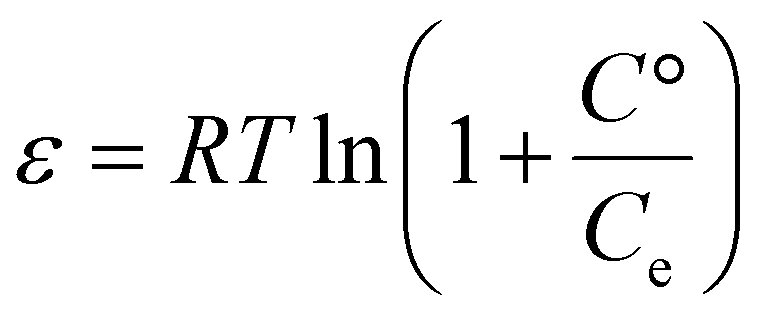	*β* (mol^2^ J^−2^)		
Sips (Langmuir–Freundlich)	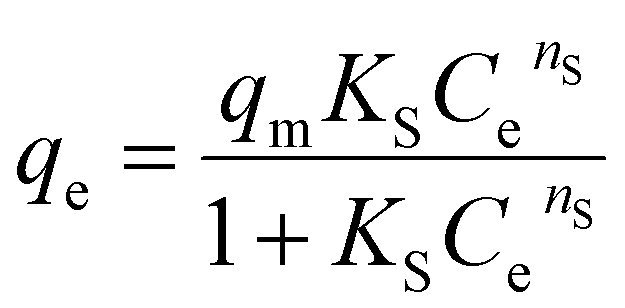	*q*_m_ (mg g^−1^)	Langmuir integrated over a quasi-Gaussian energy distribution	Freundlich at low *C*_e_; Langmuir at high *C*_e_; *n*_S_ = 1 → Langmuir
		*K*_S_ [(L mg^−1^)^*n*_S_^]		
		*n*_S_(−)		
Redlich–Peterson	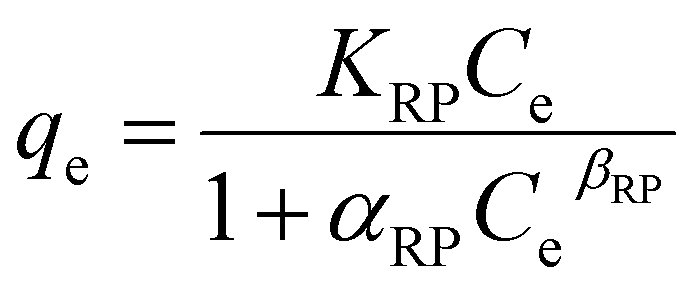	*K*_RP_ (L g^−1^)	Hybrid form interpolating between the Henry and Freundlich limits	*β*_RP_ = 1 → Langmuir; *β*_RP_ = 0 → Henry
		*α*_RP_ [(L mg^−1^)^*β*_RP_^]		
		*β*_RP_ (—)		
Toth	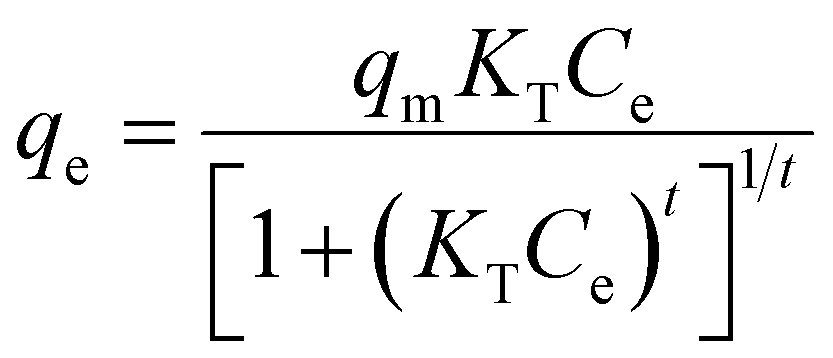	*q*_m_ (mg g^−1^)	Asymmetric quasi-Gaussian energy distribution; corrects Langmuir at both extremes	*t* = 1 → Langmuir; *t* < 1 indicates heterogeneity
		*K*_T_ (L mg^−1^)		
		*t* (—)		
Hill	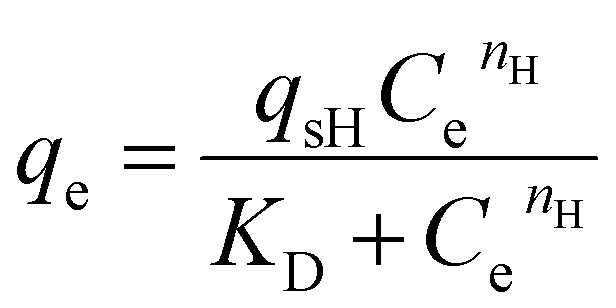	*q*_sH_ (mg g^−1^)	Cooperative binding: occupancy of one site alters the affinity of its neighbours	*n*_H_ > 1 positive cooperativity; *n*_H_ = 1 → Langmuir
		*K*_D_ [(mg L^−1^)^*n*_H_^]		
		*n*_H_ (—)		
Khan	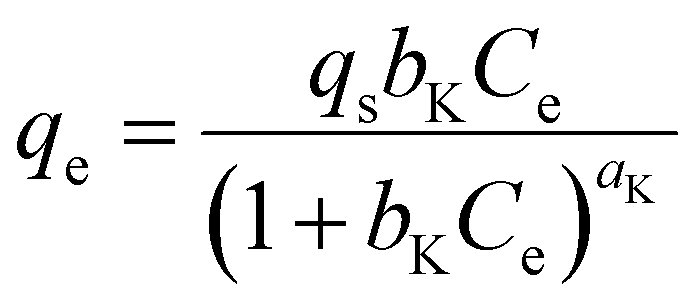	*q*_s_ (mg g^−1^)	Generalised form for multicomponent and dilute systems	*a*_K_ = 1 → Langmuir; *a*_K_ = 0 → Henry
		*b*_K_ (L mg^−1^)		
		*a*_K_ (—)		
Radke–Prausnitz	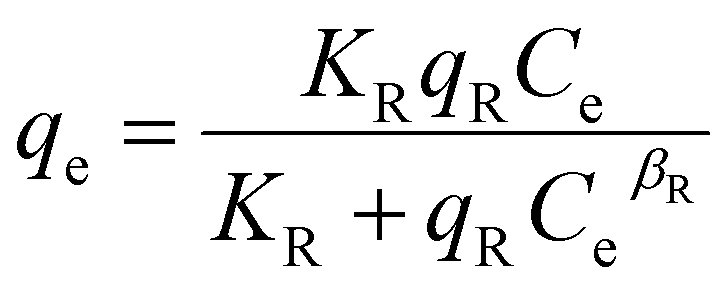	*K*_R_, *q*_R_	Dilute-solution form for heterogeneous surfaces	Henry at low loading; Freundlich at high loading
		*β*_R_ (—)		
		Units of *K*_R_ and *q*_R_ set by *β*_R_		
Brouers–Sotolongo	*q*_e_ = *q*_m_{1 − exp[–(*KC*_e_)^*α*^]}	*q*_m_ (mg g^−1^)	Lévy-distributed site-energy spectrum (deformed exponential)	*α* = 1 → Jovanovic; sharp rise then firm plateau
		*K* (L mg^−1^)		
		*α* (—)		
Jovanovic	*q*_e_ = *q*_m_[1 − exp(−*KC*_e_)]	*q*_m_ (mg g^−1^)	Monolayer with allowance for mechanical contact between adsorbate and surface	Approaches Langmuir at low coverage
		*K* (L mg^−1^)		

a Symbols: *q*_e_ (mg g^−1^), equilibrium uptake; *C*_e_ (mg L^−1^), equilibrium concentration; *R* = 8.314 J mol^−1^ K^−1^; *T* (K), absolute temperature; *C*^°^ = 1 mol L^−1^, reference concentration that makes the logarithmic argument of the Dubinin–Radushkevich potential dimensionless (*C*_e_ then in mol L^−1^); (—), dimensionless. Equation numbers refer to Section 6.1.

#### 6.1.1. Two-parameter models

The Langmuir equation remains the reference. It was derived for the chemisorption of a gas on an energetically uniform plane and rests on four assumptions: a fixed number of localised sites of identical adsorption enthalpy; strictly monolayer coverage, one adsorbate per site; no lateral interaction between occupied sites; and dynamic equilibrium between adsorption and desorption.^101^ In the liquid phase these assumptions are extended by a fifth, usually tacit one—that the solvent participates only through a constant activity—which is a poor description of an aqueous polyelectrolyte gel whose swelling state itself varies with ionic strength:1
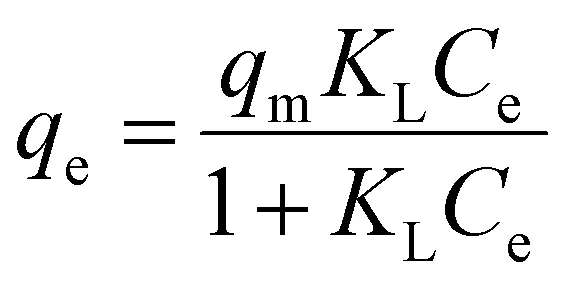
Here *q*_m_ (mg g^−1^) is the monolayer capacity and *K*_L_ (L mg^−1^) the affinity constant, the ratio of the adsorption and desorption rate constants. The dimensionless separation factor *R*_L_, eqn (2), where *C*_0_ (mg L^−1^) is the initial adsorbate concentration, provides a compact statement of favourability: *R*_L_ = 0 denotes an irreversible isotherm, 0 < *R*_L_ < 1 a favourable one, *R*_L_ = 1 a linear one and *R*_L_ > 1 an unfavourable one.^102^ Langmuir behaviour dominates both the dataset of Table 1 and the wider composite literature,^19,51^ but this fitting preference does not independently establish a site-limited surface or chemisorption. Two caveats deserve emphasis. First, *q*_m_ is an extrapolated asymptote rather than a measured quantity, and its numerical value is conditioned by the concentration interval over which the isotherm was determined; the relative contribution of this effect cannot be quantified from the present compilation.^99,103^ Second, the four Langmuir linearisations are not equivalent estimators. The type-I form, eqn (3), distributes the error differently from the type-II form, eqn (4), and the two return different parameters from the same data because linearisation transforms the error structure; non-linear regression on the untransformed equation is generally preferable when its error model is appropriate.^100,103^2
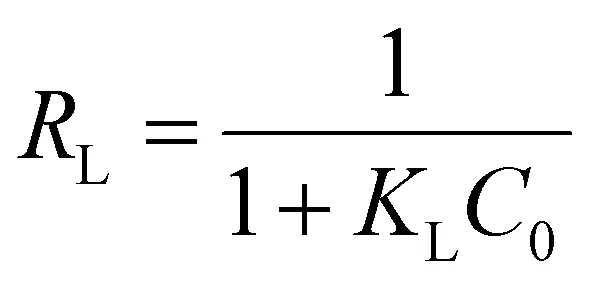
3
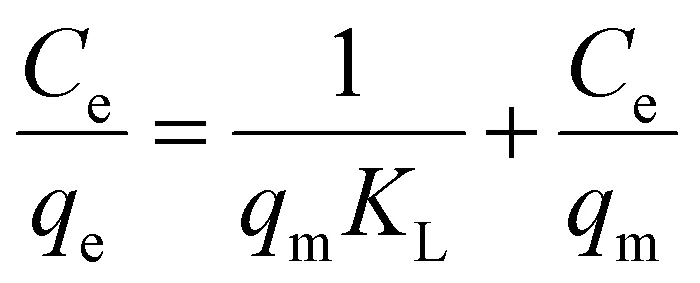
4
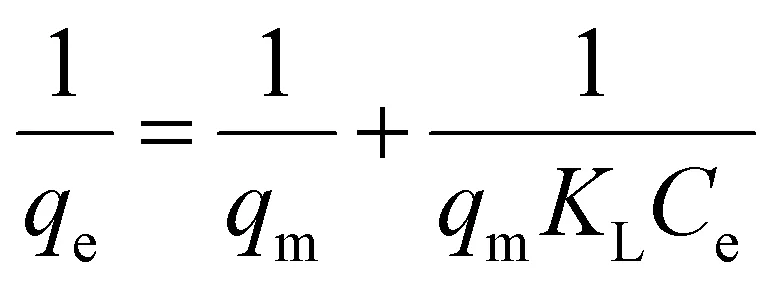


The Freundlich equation is empirical and describes adsorption on a surface whose site energies follow an approximately exponential distribution:^104^

5 *q*_e_ = *K*_F_*C*_e_^1/*n*^

*K*_F_ [(mg g^−1^)(L mg^−1^)^1/*n*^] indexes capacity and 1/*n* (dimensionless) indexes heterogeneity, with values between zero and unity denoting favourable adsorption and departure from unity increasing with surface heterogeneity.^100,102^ The model has no saturation asymptote, so a Freundlich fit that extends to the highest concentrations investigated should be read as evidence that saturation was not approached rather than as evidence of unbounded capacity. Freundlich behaviour recurs precisely where several chemically distinct site populations coexist: in the chitosan–Al_2_O_3_ hydrogel, where polymer amine sites and amphoteric oxide hydroxyls act in parallel;^56^ in the nanocellulose-containing chitosan/alginate composite;^76^ and in the nanochitosan/alginate/microcrystalline cellulose beads.^93^

The Temkin equation, eqn (6), abandons the assumption of an invariant adsorption enthalpy and postulates instead that the enthalpy of adsorption of all molecules in a layer falls linearly with coverage as a consequence of adsorbate–adsorbate repulsion.^105^ The parameter *b*_T_ (J g mol^−1^ mg^−1^) is the Temkin constant and *A*_T_ (L mg^−1^) the Temkin equilibrium binding constant, and *B* = *RT*/*b*_T_ carries the units of *q*_e_; *R* (8.314 J mol^−1^ K^−1^) is the gas constant and *T* (K) the absolute temperature. Its use in liquid-phase work is largely diagnostic; the physical situation it describes—a lateral interaction that is significant but linear in coverage—is rarely verified independently.6
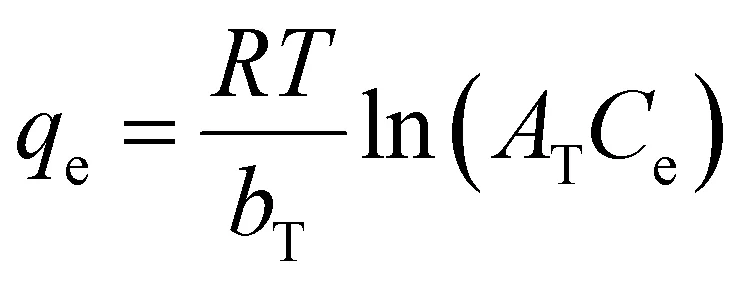


The Dubinin–Radushkevich equation is listed in Table 4.^106,107^ Its micropore-filling origin and sensitivity to the dimensionless definition of the adsorption potential limit mechanistic interpretation at a solid-solution interface. Conventional numerical energy thresholds are not reliable standalone tests of physisorption, ion exchange or chemisorption.

#### 6.1.2. Three-parameter and generalised models

Sips, Redlich–Peterson and Toth include Langmuir-like limits, but extra parameters may improve fit without providing new mechanistic evidence. Langmuir tends to Henry behaviour at low concentration; it does not generally tend to Freundlich behaviour with a non-unit exponent. Parameter identifiability, uncertainty and residual structure should be assessed before interpreting an additional fitting parameter.

The Sips or Langmuir–Freundlich equation, eqn (7), was obtained by integrating the Langmuir isotherm over a quasi-Gaussian distribution of adsorption energies.^108^ It reduces to Freundlich behaviour at low concentration and to Langmuir behaviour at high concentration, so it retains a finite saturation capacity while accommodating energetic heterogeneity—the combination that makes it appropriate for a polyelectrolyte complex bearing amine, carboxylate and, where a filler is present, oxide sites simultaneously. It describes the chitosan-in-alginate beads^72^ and the alginate–chitosan composite of Hamza *et al.*^73^ The Redlich–Peterson equation, eqn (8), interpolates between the Henry limit at *β*_RP_ = 0 and the Langmuir limit at *β*_RP_ = 1 (ref. 109) and describes the chitosan/calcium alginate/bentonite hydrogel.^71^ The Toth equation, eqn (9), was constructed specifically to correct the systematic deviation of the Langmuir form at both concentration extremes, and its exponent *t* is a direct index of heterogeneity, unity denoting a homogeneous surface.^110^7
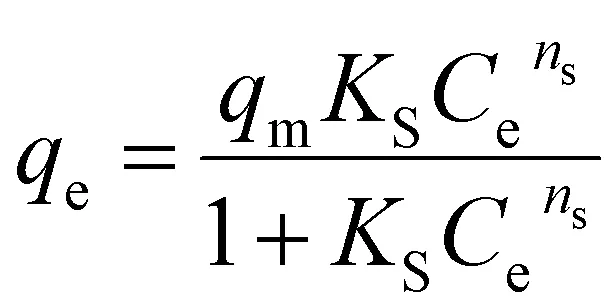
8
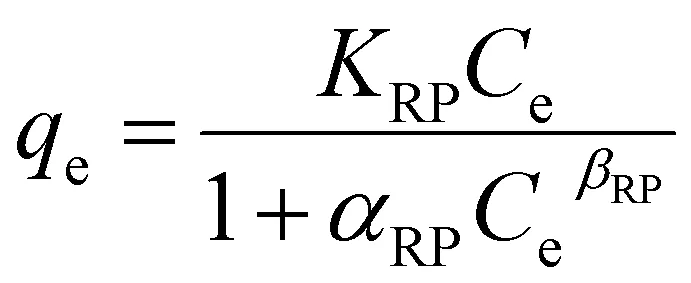
9
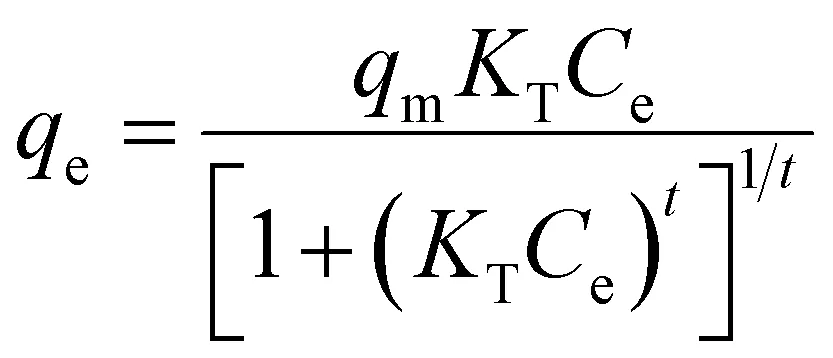


Four further equations are encountered less often but are diagnostically useful. The Hill equation, eqn (10), was derived for cooperative ligand binding and is the appropriate form when occupancy of one site alters the affinity of its neighbours;^111^ a Hill coefficient above unity indicates positive cooperativity, which in a polyelectrolyte gel would correspond to a conformational rearrangement that exposes further binding sites on loading—a plausible but rarely tested proposition for the egg-box network. The Khan equation, eqn (11), is a generalised form for multicomponent systems that reduces to Langmuir at *a*_K_ = 1.^112^ The Radke–Prausnitz equation approaches Henry behaviour at low loading and Freundlich behaviour at high loading and is particularly suited to dilute solutions.^113^ The Brouers–Sotolongo equation, eqn (12), derives from a Lévy-distributed site-energy spectrum and reduces to the Jovanovic form at *α* = 1;^114,115^ its deformed-exponential shape captures the sharp initial rise followed by a firm plateau that characterises strongly chelating sorbents.10
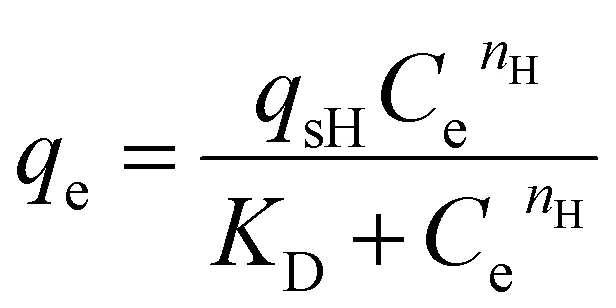
11
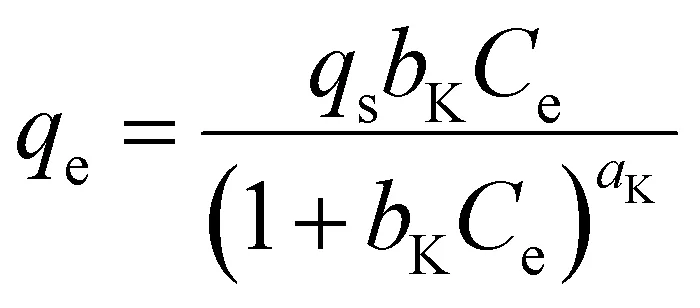

12 *q*_e_ = *q*_m_{1 − exp[−(*KC*_e_)^*α*^]}

An energy-distribution analysis may provide a model-dependent description of heterogeneity.^116^ Assigning distribution modes to electrostatic binding or chelation requires independent evidence and cannot be inferred uniquely from an isotherm derivative.

Model discrimination should combine residual diagnostics, an explicit measurement-error model, parameter uncertainty and suitable complexity penalties such as AIC or AICc.^99,100,103^ Adjusted *R*^2^ alone does not establish model validity. Giles classification provides a qualitative description of shape,^117^ rather than proof of a molecular mechanism.

### 6.2. Adsorption kinetics and rate control

Kinetic analysis addresses the rate rather than the extent of uptake, and the distinction between a rate law and a rate-controlling mechanism must be maintained throughout. Uptake on a porous sorbent proceeds through four consecutive resistances: transport through the bulk solution, diffusion across the hydrodynamic boundary layer surrounding the particle, intraparticle diffusion through the pore network, and the surface reaction itself. Bulk transport is eliminated by adequate agitation; the remaining three are what kinetic modelling attempts to separate. The inferential limits of these analyses are summarised in Fig. 10, and the corresponding equations are collected in Table 5.

**Fig. 10:**
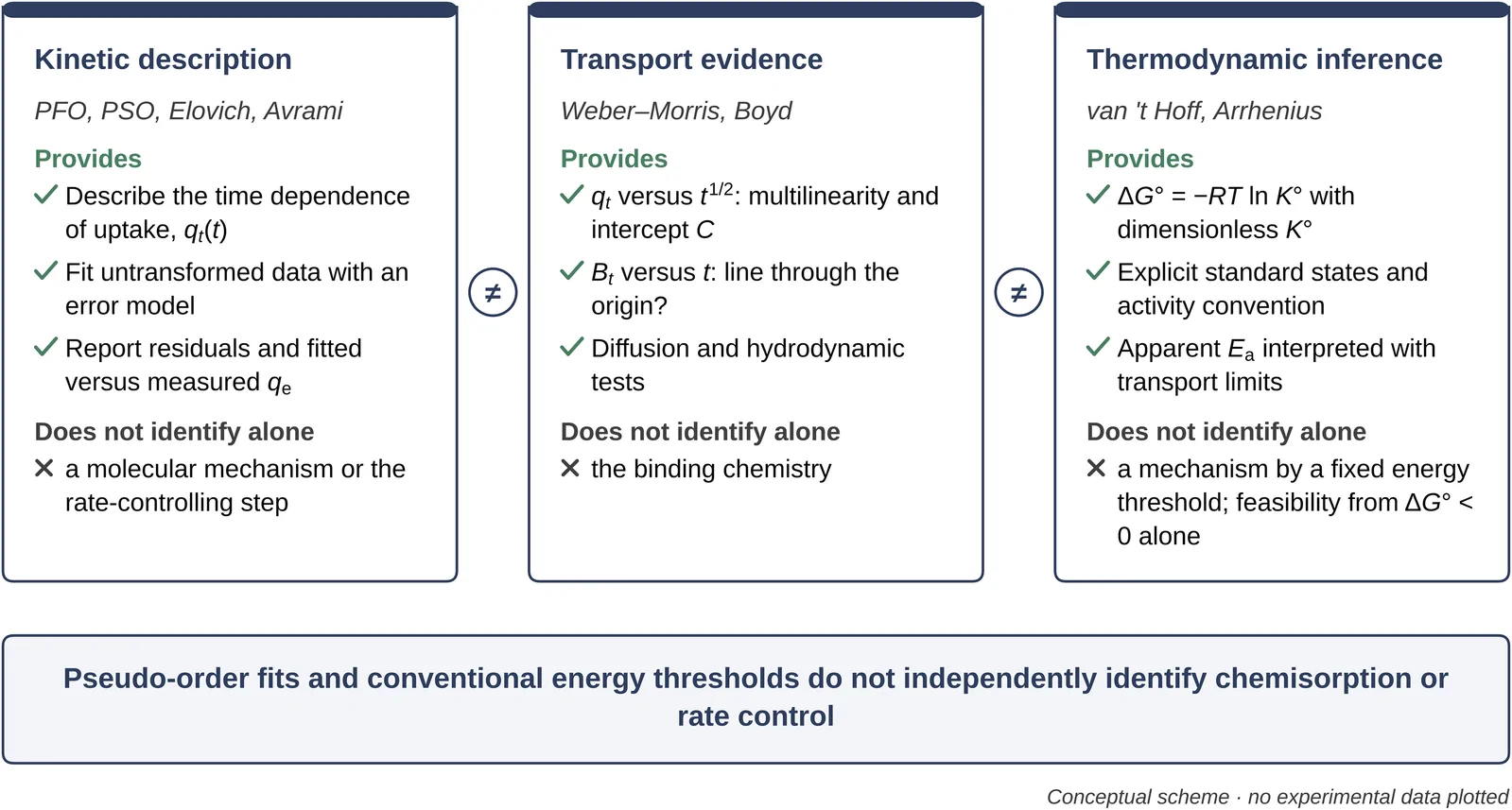
Separation of kinetic description, transport evidence and thermodynamic inference. Pseudo-order fits and conventional energy thresholds do not independently identify chemisorption or rate control. Thermodynamic calculations require a dimensionless equilibrium constant and defined standard states.

**Table 5:** Kinetic, diffusional and thermodynamic models: equation, linearised form where one is customarily used, and the quantity the analysis is intended to recover^a^

Model	Differential or integrated form	Linearised form	Quantity recovered
Pseudo-first-order (Lagergren)	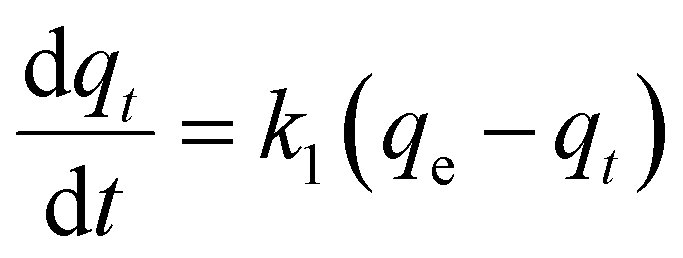	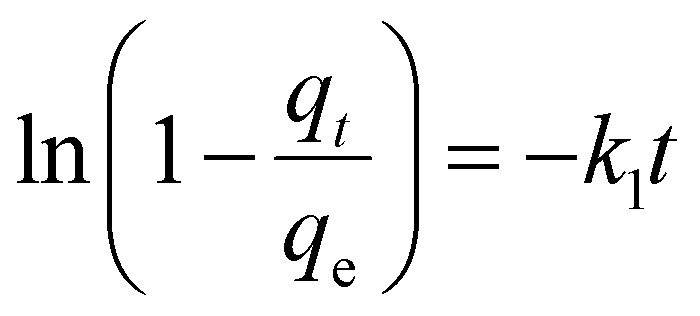	*k*_1_ (min^−1^); empirical kinetic descriptor
Pseudo-second-order (Ho–McKay)	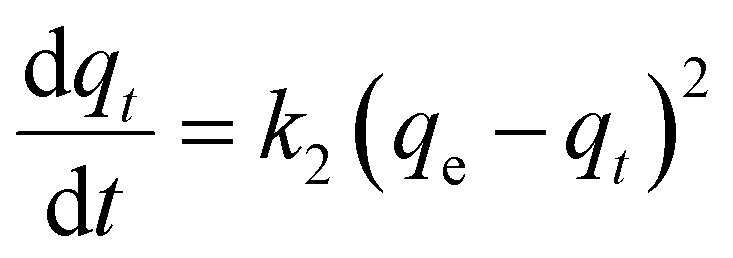	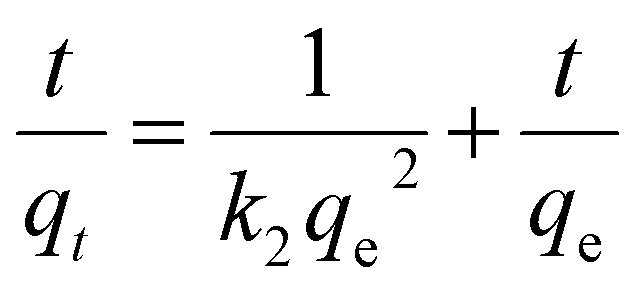	*k*_2_ (g mg^−1^ min^−1^) and the initial rate *h* = *k*_2_*q*_e_^2^ (mg g^−1^ min^−1^)
Elovich	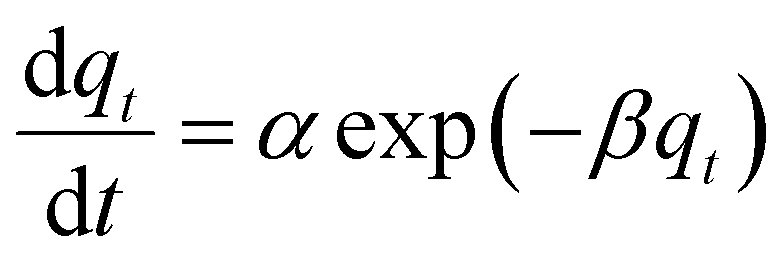	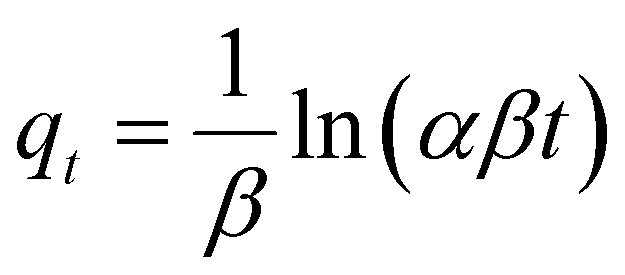	*α* (mg g^−1^ min^−1^), *β* (g mg^−1^); empirical descriptor of uptake on a heterogeneous surface; linear form valid for *αβt* ≫ 1
Avrami (fractional order)	*q*_*t*_ = *q*_e_{1 − exp[–(*k*_Av_*t*)^*n*_Av_^]}	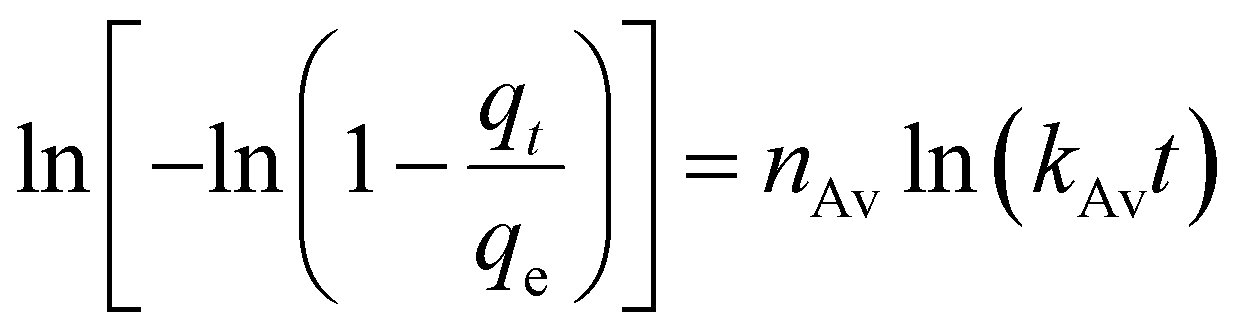	*k*_Av_ (min^−1^), *n*_Av_; a non-integer reaction order
Weber–Morris intraparticle diffusion	*q*_*t*_ = *k*_id_*t*^1/2^ + *C*	Plot *q*_*t*_ against *t*^1/2^	*k*_id_ (mg g^−1^ min^−1/2^); *C* > 0 (mg g^−1^) signals a boundary-layer contribution
Boyd	*B*_*t*_ = −0.4977 − ln(1 − *F*)	Plot *B*_*t*_ against *t* (valid for *F* > 0.85)	Line through the origin → intraparticle control
	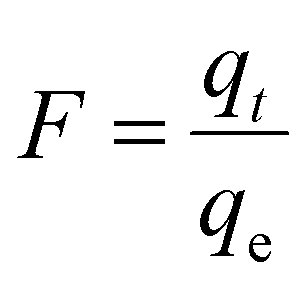		
Arrhenius	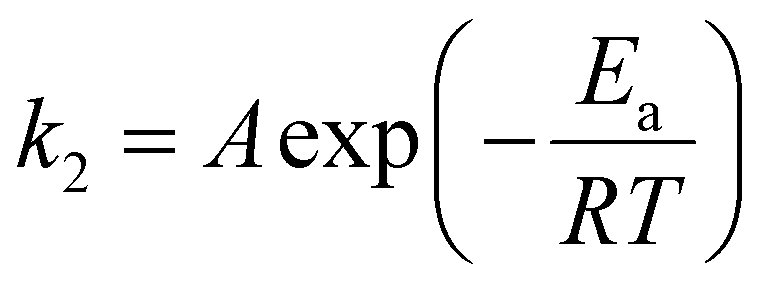	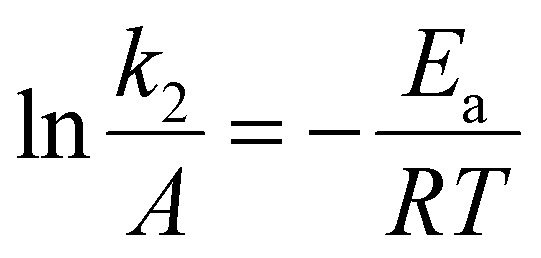	Apparent *E*_a_ (kJ mol^−1^); no universal mechanistic threshold
van't Hoff	Δ*G*° = −*RT* ln *K*	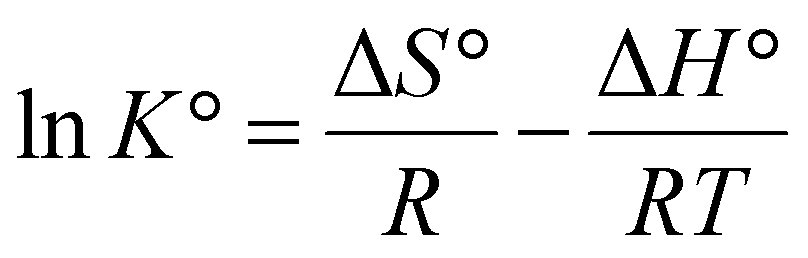	Δ*H*°, Δ*S*°; *K*° must be dimensionless

a Symbols: *q*_*t*_ (mg g^−1^), uptake at time *t* (min); *F* = *q*_*t*_/*q*_e_, fractional attainment of equilibrium; *A*, pre-exponential factor (units of *k*_2_); *E*_a_, apparent activation energy; *K*°, dimensionless standard equilibrium constant.

Pseudo-first-order and pseudo-second-order equations describe the time dependence of uptake, as summarised in Table 3.^118–121^ Neither model uniquely identifies a molecular mechanism or the rate-controlling step. The near-universal success of the pseudo-second-order fit in this literature has a statistical component: in the linearised plot of *t*/*q*_*t*_ against *t*, the term *t/q*_e_ dominates at long times, so the regression is well conditioned and can return *R*^2^ above 0.99 even for data that the model does not describe.^120,121^ Fits should use the untransformed observations with an appropriate error model and report residuals and agreement between fitted and measured equilibrium loading. Elovich behaviour is likewise a descriptive result, not independent proof of chemisorption.^122^

None of these expressions identifies the physical step that limits the rate, because all three are reaction-order laws written in the loading rather than in a concentration gradient. The Weber–Morris expression, eqn (13), in which *q*_*t*_ (mg g^−1^) is the uptake at time *t* (min), *k*_id_ (mg g^−1^ min^−1/2^) the intraparticle diffusion rate constant and *C* (mg g^−1^) the intercept, addresses that question directly by testing the Fickian prediction that an intraparticle-diffusion-controlled uptake is linear in the square root of time.^123^ Multi-linearity rather than linearity indicates a succession of controlling resistances—external film diffusion, then intraparticle diffusion through the pore network, then equilibration on the least accessible interior sites—and an intercept *C* greater than zero establishes that intraparticle diffusion is not the sole rate-controlling step, larger values of *C* indicating a greater boundary-layer contribution. The Boyd treatment sharpens the same test: with *F* = *q*_*t*_/*q*_e_ (dimensionless fractional attainment of equilibrium), the quantity *B*_*t*_ is given by eqn (14) for *F* above 0.85, and a plot of *B*_*t*_ against *t* that is linear and passes through the origin identifies intraparticle diffusion as rate-controlling, whereas a non-zero intercept implicates film diffusion.^124^ For hydrogel and aerogel composites this analysis is particularly informative, because the hierarchical porosity generated by freeze-drying is intended precisely to shorten the intraparticle path;^70,78^ a Weber–Morris plot whose second stage retains a steep slope is the direct evidence that the intention was realised.

13 *q*_*t*_ = *k*_id_*t*^1/2^ + *C*

14
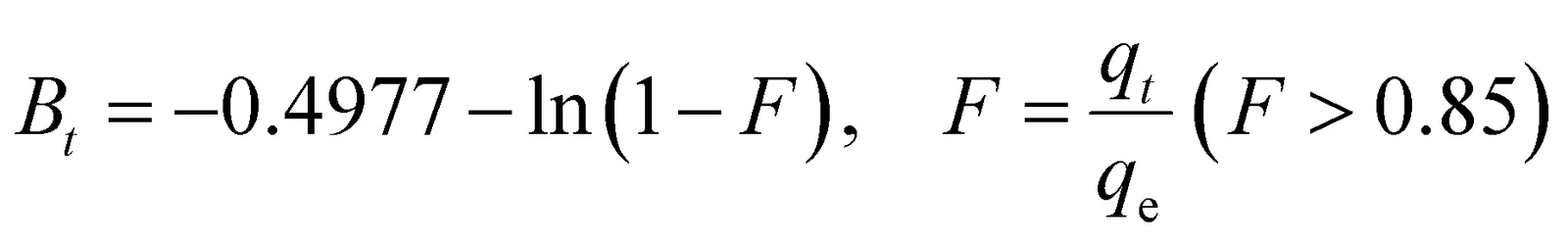


Thermodynamic analysis uses eqn (15) and (16), where Δ*G*° and Δ*H*° (kJ mol^−1^) and Δ*S*° (J mol^−1^ K^−1^) are the standard Gibbs energy, enthalpy and entropy changes and *K*° is dimensionless and the standard states are explicit.^125–128^ A dimensional distribution or Langmuir constant cannot be inserted directly into the logarithm. A valid conversion requires an explicitly defined adsorption equilibrium and activity convention, rather than an automatic multiplication by water concentration. Negative Δ*G*° describes the chosen standard-state equilibrium and does not alone predict process feasibility. Apparent Arrhenius activation energies from fitted kinetic constants must be interpreted with transport and model limitations; fixed numerical thresholds do not establish a mechanism.

15 Δ*G*° = −*RT* ln *K*°

16
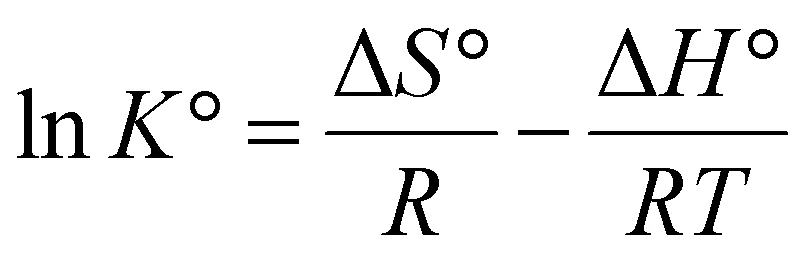


### 6.3. Adsorption mechanism

The physical and chemical properties of biopolymer-based adsorbents, and hence their interaction with metal ions, are governed by solution pH and by the other constituents of the system.^19^ Five mechanisms operate on chitosan–alginate composites, ordinarily in combination rather than in isolation; they are set out in Fig. 11, together with the evidence required to establish each.

**Fig. 11:**
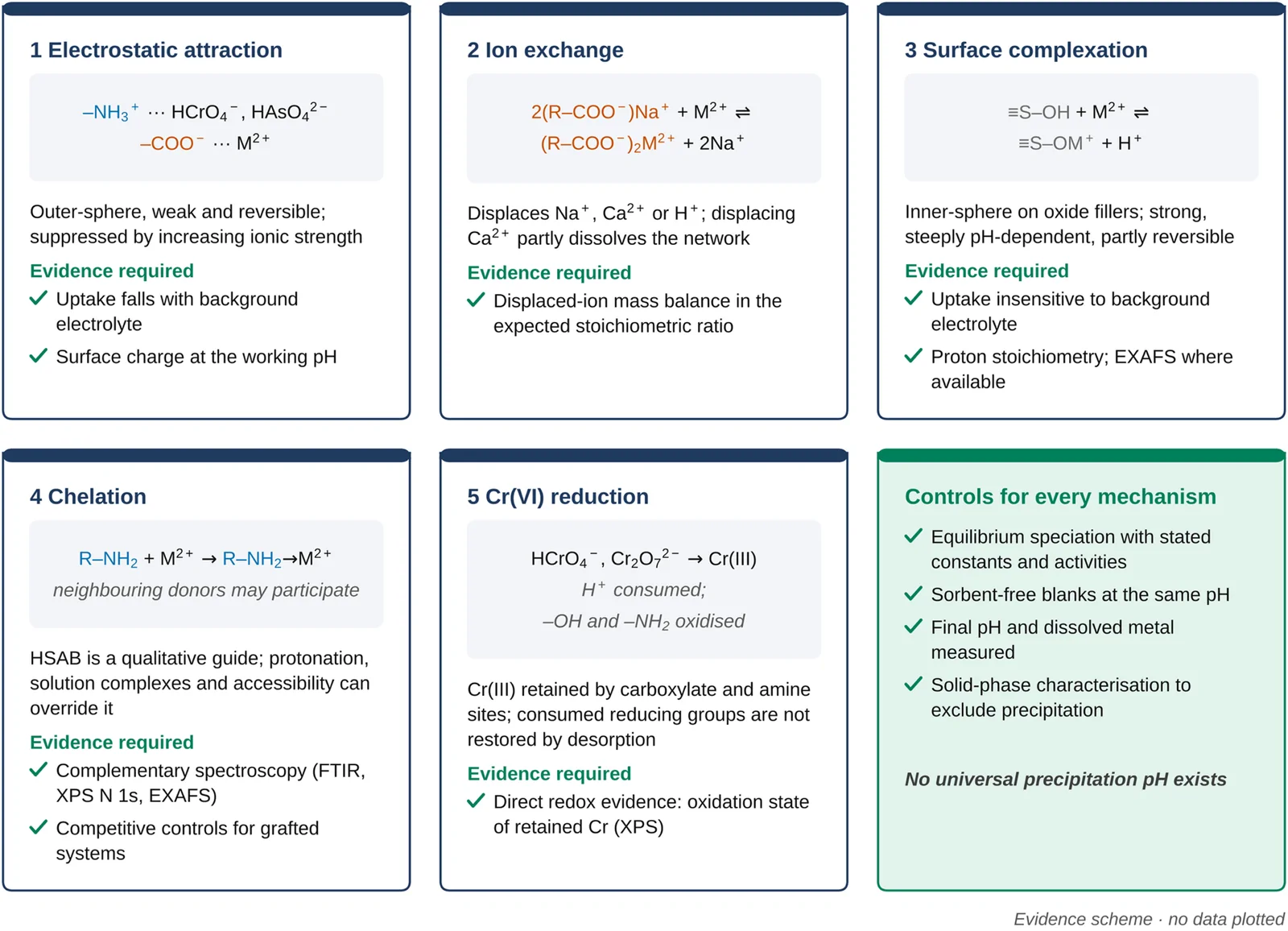
Mechanistic evidence requirements for electrostatic attraction, ion exchange, surface complexation, chelation and Cr(vi) reduction. Speciation and precipitation controls are needed alongside spectroscopy; there is no universal precipitation pH.

Electrostatic attraction is the first and the most directly pH-controlled. Above the point of zero charge, deprotonation of the alginate carboxyl groups and of the surface hydroxyls of any inorganic phase generates a net negative surface that accumulates metal cations in the diffuse layer; below the p*K*_a_ of the glucosamine amine, protonation to –NH_3_^+^ generates a net positive surface that attracts chromate, dichromate and arsenate. The interaction is outer-sphere: the cation retains its primary hydration shell and is held by long-range coulombic forces, so the binding is comparatively weak, is strongly suppressed by increasing ionic strength, and is readily reversed. This is why the same composite family is operated at pH 5–6 for Pb^2+^, Cu^2+^ and Cd^2+^ and at pH 2–4 for Cr(vi), as the pH column of Table 1 shows ref. 56, 69 and 79. Metal hydrolysis and precipitation may occur within this same operating range. The relevant threshold must be evaluated for each solution; sorbent-free controls and dissolved-metal balances are necessary to distinguish removal from adsorption.^97,98^

Ion exchange follows from the counterions that a polyelectrolyte network necessarily carries. Calcium ions occupying the egg-box junctions of alginate, and the sodium ions or protons associated with free carboxylate groups, are displaced stoichiometrically by incoming metal cations according to 2(R–COO^−^)Na^+^ + M^2+^ ⇌ (R–COO^−^)_2_M^2+^ + 2Na^+^. The mechanism is distinguished from simple electrostatic accumulation by a testable prediction—the appearance of the displaced ion in solution in the expected stoichiometric ratio—and it was identified as the dominant route in magnetic alginate/ternary metal hydrogel composites.^19^ Because the egg-box junction is itself a chelate, the affinity sequence for exchange follows the stability of that junction rather than simple charge density, and displacement of Ca^2+^ by a metal of higher affinity is simultaneously an adsorption event and a partial dissolution of the network.^45^

Surface complexation operates wherever an inorganic phase is present. Amphoteric surface hydroxyls, written generically as ≡S–OH, undergo two protonation equilibria whose intrinsic constants define the point of zero charge, and exchange ligands with metal ions according to ≡S–OH + M^2+^ ⇌ ≡S–OM^+^ + H^+^, with a bidentate analogue 2(≡S–OH) + M^2+^ ⇌ (≡S–O)_2_M + 2H^+^.^97,98^ The defining feature is that the complex is inner-sphere: the metal is bonded directly to a surface oxygen with no intervening water molecule, which makes the interaction strong, comparatively insensitive to ionic strength, and only partially reversible. The proton stoichiometry of the mass-law expression is the reason surface complexation is so steeply pH-dependent, and it is also the property that permits inner-sphere and outer-sphere binding to be distinguished experimentally without spectroscopy, by testing whether uptake responds to background electrolyte concentration. This mechanism is what the inorganic phase contributes in the TiO_2_,^85^ Fe/Mn oxide,^80^ Al_2_O_3_ (ref. 56) and Fe_3_O_4_ (ref. 77 and 92) composites, and its pH dependence is that of the oxide surface rather than that of the polymer—which is precisely why a hybrid operates over a wider pH range than either component.

Chelation involves coordination by accessible amino groups and, where geometry permits, neighbouring donors. Hard–soft acid–base concepts provide a qualitative design guide: Hg^2+^ and Cd^2+^ are soft acids, whereas Pb^2+^ is commonly treated as borderline.^129^ Ligand protonation, solution complexes and network accessibility can override a simple donor-based preference. The Irving–Williams sequence describes a general trend for divalent first-row transition-metal complexes;^130^ without a chitosan-specific validation it is not asserted to predict selectivity in these composites. The grafted systems in ref. 60, 74, 78, 79, 84, 88 and 94 must be assessed using their own competitive controls.

Reduction accompanies adsorption in the specific case of Cr(vi). Hexavalent chromium, present as HCrO_4_^−^ or Cr_2_O_7_^2−^ in acidic media, is reduced to the far less toxic and far less mobile Cr(iii) at the sorbent surface, the hydroxyl and amino groups of the polysaccharide acting as electron donors and being oxidised in the process; the Cr(iii) so produced is then retained by complexation with carboxylate and amine sites. The half-reaction consumes protons, which accounts for the strongly acidic optimum reported for every Cr(vi) sorbent in Table 1 and for the loss of performance as the pH rises. The mechanism has been established by X-ray photoelectron spectroscopy in chitosan and alginate systems.^19,43^ It also carries an operational consequence that is seldom drawn: because the reducing groups are consumed, the capacity of a Cr(vi) sorbent is not fully restored by desorption alone, and cycling data for Cr(vi) should not be compared with cycling data for divalent cations.

Establishing which of these mechanisms operates requires spectroscopic rather than isotherm evidence, and the discriminating criteria are specific. Fourier-transform infrared spectroscopy identifies the groups involved through shifts in the amide I and II bands, in the broad O–H/N–H envelope near 3400 cm^−1^, and—most usefully—in the separation Δ*ν* = *ν*_as_(COO^−^) − *ν*_s_(COO^−^), whose change relative to the free carboxylate can help assess monodentate from bidentate chelating and bridging coordination;^131^ the interaction between alginate and chitosan themselves has been characterised in exactly this way.^52^ X-ray photoelectron spectroscopy resolves the oxidation state of the bound metal, and can provide evidence for the Cr(vi) → Cr(iii) conversion directly; the binding-energy shift of the N 1s level on coordination of the amine, and the deconvolution of the O 1s envelope into C–O, C=O and metal–O contributions, together distinguish coordination from mere electrostatic accumulation.^19^ Extended X-ray absorption fine structure spectroscopy provides complementary local-structure evidence, resolving the metal–oxygen distance and coordination number and thereby separating inner-sphere from outer-sphere complexes, although it remains rare in this literature. Scanning electron microscopy with energy-dispersive analysis confirms whether uptake is confined to an outer shell or penetrates the bead, X-ray diffraction reveals whether a crystalline precipitate contributes to removal; absence of a diffraction peak does not exclude an amorphous precipitate, and nitrogen physisorption quantifies the accessible surface area and pore structure.^53,54^ Molecular imprinting studies provide the clearest structure–selectivity evidence available for this class of material, since the selectivity is by construction attributable to cavity geometry.^49^ The characterisation strategy is summarised in Fig. 6.

### 6.4. Regeneration and reusability

Regeneration determines whether a sorbent constitutes a technology or a laboratory result. Two criteria must be satisfied simultaneously: the metal must be desorbed efficiently enough to restore capacity, and the network must survive the desorption chemistry well enough to be reused. Materials that satisfy the first but not the second are common, and the literature reports the first far more often than the second.^132,133^ The principal regeneration routes and the reporting gaps that persist are summarised in Fig. 12.

**Fig. 12:**
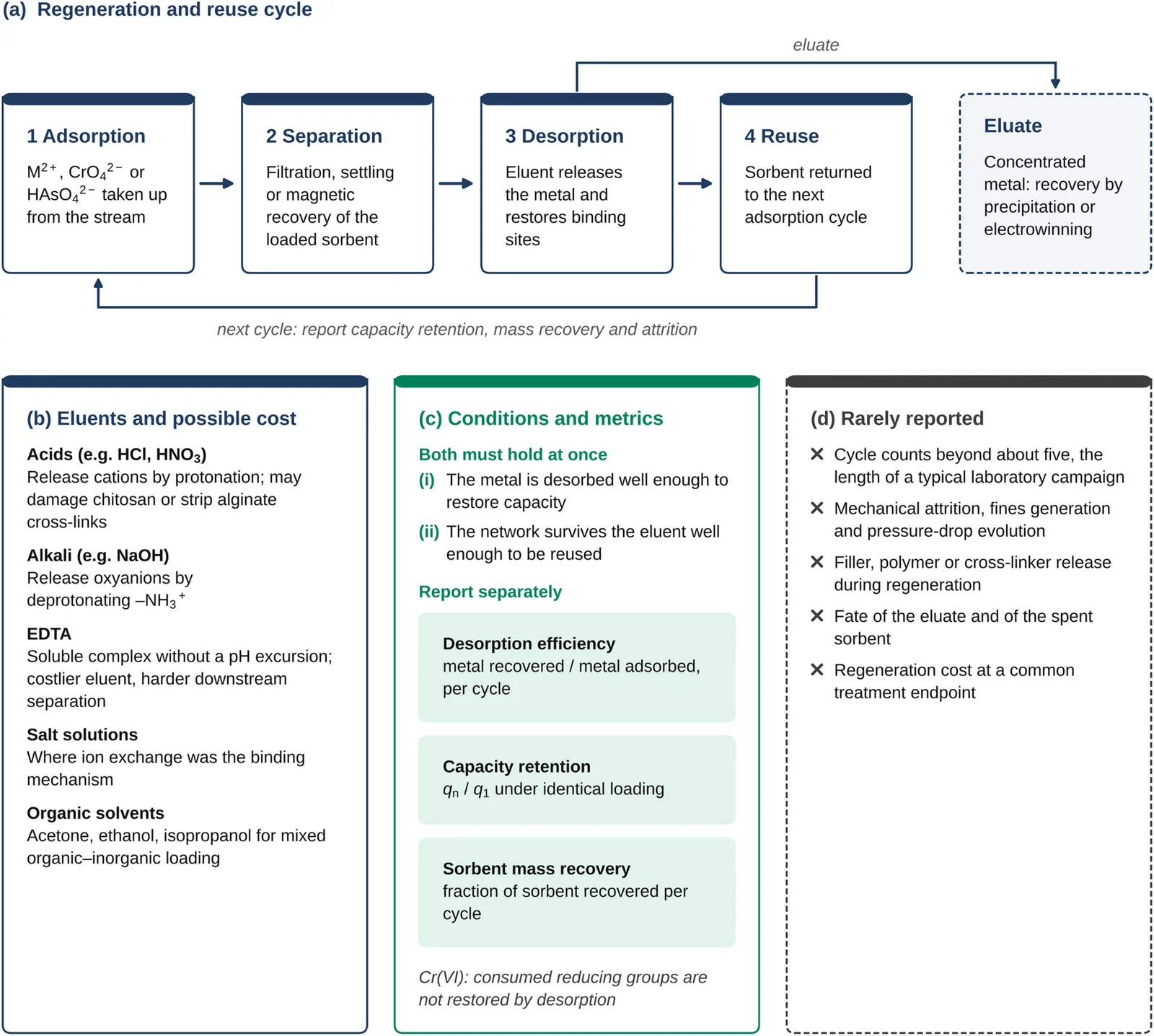
Regeneration of biopolymeric composite adsorbents. (a) Adsorption–separation–desorption–reuse cycle, with metal recovery from the eluate; (b) eluent classes and their possible cost to the network; (c) the two conditions that regeneration must satisfy simultaneously and the distinct metrics used to report it; (d) aspects of cycling performance that are rarely reported.

Acid eluents protonate binding groups and can release adsorbed cations, but may also damage chitosan networks or remove alginate cross-linking ions.^132,133^

Alkaline elution is used for anionic species, where raising the pH deprotonates the amino groups and releases electrostatically bound chromate or arsenate. Complexing agents such as EDTA offer a gentler alternative for cations, forming soluble metal complexes without the pH excursion, at the cost of a more expensive eluent and a more complex downstream separation. Salt solutions regenerate by ion exchange where that was the binding mechanism, and organic solvents including acetone, ethanol and isopropanol are used for mixed organic–inorganic loading.^19,132^

Results for chitosan-only or alginate-only composites cannot be presented as hybrid regeneration evidence. For example, calcium-cross-linked alginate hydrogels containing algal biochar are reported to remove Cu^2+^ (136 mg g^−1^) and Fe^3+^ (1.25 mg g^−1^) at pH 4 and 298 K and to remain active after three adsorption–regeneration cycles;^134^ this single-polymer result does not document hybrid behaviour, the published abstract gives no capacity-retention value, and Fe^3+^ uptake at pH 4 would require a precipitation check (Section 5.3). Ref. 93 includes adsorption–desorption studies on a hybrid formulation, but a common cross-study retention range is not inferred. Report desorption efficiency as the fraction of the adsorbed metal recovered in a cycle, capacity retention as uptake in cycle *n* divided by uptake in the first cycle under identical loading conditions, and sorbent mass recovery separately. Removal efficiency, desorption efficiency and retained capacity have different denominators.

Three practical points follow. First, the number of cycles reported is usually five, which is far short of what a fixed-bed installation requires and which reflects the duration of a laboratory campaign rather than any property of the material. Second, capacity retention alone is an incomplete criterion: mechanical attrition, fines generation and pressure-drop evolution determine column life at least as strongly, and they are almost never reported. Third, the eluate is a concentrated metal solution and therefore a resource as much as a waste; recovery by precipitation or electrowinning converts a treatment cost into a partial credit, and the possibility of valorising the spent sorbent itself has been explored for biopolymer-based materials.^133^ Regeneration should be designed into the material at the synthesis stage, through cross-link density and eluent compatibility, rather than investigated after the fact.

### 6.5. Operational comparison and translation to treatment

Fixed-bed evidence should report bed dimensions, dry sorbent mass, particle size, influent composition and concentration, flow, empty-bed contact time, breakthrough criterion, exhaustion criterion, pressure drop and integrated metal balance. Dynamic working capacity must be calculated from the area between influent and effluent concentration curves. Batch and column studies are included in ref. 95, but column performance cannot be inferred from a batch isotherm. In a fixed-bed test of EDTA–chitosan/alginate beads (0.5 g dry mass; bed height 18 cm; internal diameter 0.7 cm) fed with 25 mg per L Pb(ii) at 1.5 mL min^−1^, breakthrough (*C*_*t*_/*C*_0_ = 0.05) occurred after about 120 min and exhaustion (*C*_*t*_/*C*_0_ = 0.95) after about 585 min, giving a breakthrough capacity of 20.92 mg g^−1^.^74^ This dynamic capacity is less than 5% of the 504.11 mg g^−1^ equilibrium maximum reported for the same beads in batch; it was obtained with a single-solute synthetic feed and does not describe operation in a real effluent. In a fixed-bed test of a magnetic calcium alginate/carboxymethyl chitosan powder fed with a ternary Nd(iii)/Tb(iii)/Dy(iii) solution (30 mg L^−1^ each; 0.3 g sorbent mixed with sand; bed height 6.5 cm; internal diameter 0.5 cm; 1 mL min^−1^), breakthrough (*C*_*t*_/*C*_0_ = 0.05) occurred after about 95, 105 and 120 min, with breakthrough-curve capacities of 22.70, 22.70 and 25.54 mg g^−1^ and a column utilisation below 50%;^95^ this feed was also synthetic. Fibrous formats are a possible route to low-pressure-drop contactors: electrospun alginate–*Moringa oleifera* fibres^135^ and wet-spun cellulose–*M. oleifera* fibres^136^ have been produced, and alginate–cellulose–*M. oleifera* composites have been reviewed.^137^ In the two primary studies, however, metal adsorption was either not tested or assessed only qualitatively by SEM-EDX, so their dynamic performance remains to be established.

Multi-metal tests should provide component-resolved equilibrium and kinetic data with competing Ca^2+^, Mg^2+^, dissolved organic matter and relevant salinity. In one of the few tests on a natural water, alginate–chitosan beads (1 g L^−1^, 5 h, original pH 5.8) removed 99.44% of Cd and 99.21% of Pb from a lake-water sample containing about 1.96 mg Cd per L and 2.03 mg Pb per L together with Cu, Zn, Al, Fe, Hg and Ni and about 3905 mg L^−1^ of dissolved salts;^73^ the source describes the sample as “enriched” in Cd and Pb without stating whether these metals were added, and some residual concentrations remained above drinking-water limits. In textile effluents, dyes are additional competitors; chitosan-based adsorbents for dye removal have been reviewed separately.^138^ Evidence from ref. 60, 68, 84 and 95 should be evaluated at the actual matrix composition. No numerical selectivity order is reconstructed from missing data.

Filler containment is a separate acceptance criterion. Fe_3_O_4_-containing and ZIF-8-containing sorbents require dissolved-element and particulate-release measurements during adsorption, washing and regeneration, together with polymer or cross-linker release where relevant. Magnetic recoverability does not demonstrate absence of leaching; immobilisation does not establish hydrolytic stability. No leaching concentration is inferred from the present compilation. Nanoparticle leaching, bioaccumulation and ecological effects of biopolymer-based nanocomposites are likewise identified as open issues in a recent review.^139^

A useful economic functional unit is the cost per volume treated to a specified effluent limit, including synthesis, drying, cross-linking, separation, replacement, regeneration, eluate treatment and disposal. Life-cycle comparison should use the same treatment endpoint and include chemical and energy inputs, metal recovery and end-of-life fate. Without such inventories, no numerical cost or environmental advantage over activated carbon can be claimed.^7,20,132,133^

## 7. Research gaps and future directions

Priorities are ordered by the uncertainty they resolve, the engineering barrier they address and their relevance to continuous treatment.

### 7.1. Highest priority is reliable and traceable measurement

Complete primary full-text extraction, distinguish observed uptake from fitted capacity, report wet or dry mass basis and experimental conditions, and include uncertainty and precipitation controls. These are prerequisites for a defensible material comparison.^99,103^

### 7.2. Validate competitive uptake and network stability in relevant effluents

Measure component-resolved adsorption, dissolved and particulate filler release, polymer loss and toxicity where appropriate. This addresses selectivity and containment barriers that single-solute batch capacity cannot resolve.^60,68,84,95^

### 7.3. Establish dynamic working capacity and regeneration durability

Quantify breakthrough, pressure drop, attrition, mass recovery, desorption and capacity retention over cycling. This provides the operational basis for choosing among reinforced beads, magnetic systems and porous grafted networks.^93,95,132^

### 7.4. Evaluate cost and environmental burdens at a common treatment endpoint

Include modification chemicals, drying, regeneration, metal recovery and spent-sorbent management. This is necessary before renewable feedstock can be translated into a claim of process sustainability.^7,20,133^

### 7.5. Develop selective architectures after establishing the preceding evidence

Donor matching and imprinting are useful hypotheses,^18,49^ but additions should be justified by matched controls and competitive performance. Adding a filler does not necessarily increase uptake; the values reported in ref. 76 provide no evidence of a gain.

## 8. Conclusions

Chitosan and alginate provide complementary functionality and adaptable processing routes for metal-removal sorbents. Modification and composite formation can change affinity, stability and recoverability, but performance depends on formulation, speciation and operating conditions. The descriptive compilation does not establish a universally superior material or a causal dominance of donor chemistry over composition.

Isotherm and kinetic fits are descriptive evidence and must be separated from mechanistic assignments. Adsorption must be distinguished from precipitation, and desorption efficiency from capacity retention. Progress towards practical treatment requires traceable primary data, within-study controls, competitive-matrix validation, filler containment and dynamic regeneration performance. These conclusions introduce no new quantitative benchmark.

## Conflicts of interest

The authors declare that they have no known competing financial interests or personal relationships that could have appeared to influence the work reported in this paper.

## A. Appendix

### A.1. Appendix A

#### A.1.1. Literature search and selection record

Tables 6 and 7 document the search reported in Section 1.1. Counts are those recorded by the authors during screening; the procedure is a structured search for a narrative review, not a systematic review.

**Table 6:** Database search strings (last search 14 May 2026; no date or language filter)

Database	Field searched	Search string	Records
Scopus	TITLE-ABS-KEY	TITLE-ABS-KEY (chitosan OR “carboxymethyl chitosan” OR chitooligosaccharide*) AND alginate AND (adsorp* OR biosorp* OR sorption) AND (metal* OR metalloid* OR copper OR lead OR cadmium OR chromium OR arsenic OR mercury OR nickel OR zinc OR manganese OR selenium OR “rare earth”) AND (water OR wastewater OR aqueous OR effluent*)	310
Web of science core collection	TS (topic)	TS = (chitosan OR “carboxymethyl chitosan” OR chitooligosaccharide*) AND alginate AND (adsorp* OR biosorp* OR sorption) AND (metal* OR metalloid* OR copper OR lead OR cadmium OR chromium OR arsenic OR mercury OR nickel OR zinc OR manganese OR selenium OR “rare earth”) AND (water OR wastewater OR aqueous OR effluent*)	250

**Table 7:** Record of study identification and selection^a^

Step	Number
Records retrieved from Scopus	310
Records retrieved from web of science	250
Total before de-duplication	560
Duplicates removed	−220
Records screened on title and abstract	340
Records excluded on title and abstract	−250
Full texts sought	90
Full texts not obtained	−5
Full texts assessed	85
Publications excluded after full-text assessment	−61
Studies retained from the databases	24
Additional publications identified from reference lists	14
Additional publications excluded	−4
Additional studies retained	10
Final corpus of primary studies	34

a Exclusion criteria are those stated in Section 1.1. The 34 primary studies contribute the 51 material–metal entries of Tables 1–3; review articles used for context are not counted.

## Supplementary Material

RA-OLF-D6RA08674H-s001

## Data Availability

No new experimental data were generated. This review discusses published literature and includes a descriptive compilation in Tables 1–3. Source citations accompany the entries. Missing conditions and unresolved extraction limits are identified in Section 1.1 and the table notes; the compilation is not a normalised benchmark. The search strings and the record of study selection are given in Appendix A, and the experimental conditions checked against the full texts for 46 entries from 31 studies are given in Table S1 (supplementary information (SI)). Supplementary information: Table S1 lists the experimental conditions (pH, isotherm models, concentration range, adsorbent dose, temperature, electrolyte and mass basis) extracted from the source articles for the entries compiled in Table 1a–c, with their location in each source. See DOI: https://doi.org/10.1039/d6ra08674h.
