# Supplementary material for: Chitosan- and alginate-based biopolymeric composites for the adsorption of heavy metal ions from wastewater: modification strategies, composite architectures, adsorption behaviour and regeneration
Source: RSC Adv. 2026 Nov 11. Online ahead of print. doi: 10.1039/d6ra08674h (PMC13753780; doi:10.1039/d6ra08674h)
Supplement: Supplementary file 1 [file RA-OLF-D6RA08674H-s001.pdf]

Supplementary Information (SI) for RSC Advances.
This journal is © The Royal Society of Chemistry

Electronic Supplementary Information

Chitosan- and alginate-based biopolymeric composites for the adsorption of heavy metal ions from wastewater: modification strategies, composite architectures, adsorption behaviour and regeneration

Table S1. Experimental conditions extracted from the full texts of the primary studies in Tables 1a–c

Each row corresponds to one material–contaminant–result entry of Tables 1a–c. Conditions were read from the full text of the article: for open-access articles from the HTML version (section, table and figure numbers), for the other articles from the publisher PDF (p. = page of the PDF file). Conditions are those of the experiment from which the tabulated value derives. Reported = stated explicitly in the source; calculated = derived from stated quantities, with the formula given; n.r. = not reported in the full text consulted; n.v. = not verifiable because the required file (e.g. the article's supplementary material) was not available; ambiguous / non-determinable = the source allows more than one reading (reason given). † = model-derived capacity exceeding the largest uptake attainable from the highest initial concentration and the adsorbent dose, C0,max V/m. Supplementary files of the source articles were not consulted. 31 of the 34 studies (46 of the 51 entries) were verified in this way; the other three studies are listed below the table.

Reference (DOI)	Table 1 row	Material	Contaminant	Capacity and units	Experimental or model	pH	Isotherm model	Initial concentration range	Adsorbent dose	Temperature	Ionic strength / electrolyte	Mass basis	Location in source	Remarks
R. S. Kamal et al., ref. 56 (10.1186/s13065-025-01633-9)	1a	Chitosan-Al2O3 green hydrogel composite	Cr(VI)	48.9–51.5 mg g−1 'maximum metal uptake' (Abstract); Langmuir Qm 49.87–51.5 mg g−1 for the six hydrogels (Table 4)	non déterminable: the lower bound 48.9 does not appear in Table 4 (Langmuir Qm min. 49.87); upper bound 51.5 = Langmuir Qm of Cs1/AAm1/Gly1/Al0.05	2 (optimum; pH series 2–10, Fig. 2)	Freundlich best fit (R2 0.964–0.999); Langmuir also fitted (Table 4)	25, 50, 75 and 100 ppm Cr(VI) (Adsorption studies section)	0.1 g sorbent (tea-bag method); 100 mL stated for pH series → 1 g L−1 (volume for isotherm series not restated)	room temperature for pH/time series (figure captions); 30, 40, 50 °C in temperature series; isotherm temperature n.r.	n.r.	air-dried hydrogel discs (Preparation section) — dry basis	Abstract; Methods 'Adsorption studies'; Results 'Effect of pH' (Fig. 2); 'Kinetic isotherms' (Fig. 8, Table 4)	Single-polymer comparator (no alginate). Also reports treatment of a real textile wastewater (Table 6; Cu 131, Cr(VI) 33.8, Hg 21.17, Pb 14.18 ppm).
M. Awed et al., ref. 62 (10.1007/s42250-023-00806-0)	1a	Chitosan nanoparticles-alginate/methyl acrylate beads (CS NPs-Alg/MA)	Pb^{2+}	80.6 mg g−1 (Langmuir q_m, Table 2)	model-derived (Langmuir)	5.3 (pH 3–11 studied)	Langmuir (R2 0.9998) preferred to Freundlich (0.823)	10–200 mg L−1	0.2 g in 100 mL (2 g L−1); 120 min	room temperature	n.r.	n.r. (gel beads used after methanol washing)	p. 3 §2.2.3 (iv); p. 10 Table 2	Table 1a pH corrected from n.s. to 5.3 and model from n.s. to Langmuir.
M. T. ALSamman and J. Sánchez et al., ref. 63 (10.3390/polym15092192)	1a	Semi-IPN poly(acrylic acid)/alginate (PAA/alginate)	Cu^{2+}	63.59 mg g−1 (Abstract); 67 mg g−1 at 1000 mg L−1 (Conclusions)	experimental (no isotherm model fitted)	4.0 (optimum; pH study 2–4.5, Fig. 10B)	n.r. (no isotherm model reported)	10–1000 mg L−1 Cu(II) (§3.4.5, Fig. 13B)	0.05 g xerogel; 10 mL stated in Conclusions only → 5 g L−1 (0.05 g/0.010 L)	n.r.	n.r.	dry xerogel powder (§2.2: gels dried and ground)	Abstract; §2.2; §3.4.2 Fig. 10B; §3.4.5 Fig. 13B; §4 Conclusions	Two different values for the same material (63.59 vs 67 mg g−1); Table 1a uses 63.59. Volume given only in Conclusions.
M. T. ALSamman and J. Sánchez et al., ref. 63 (10.3390/polym15092192)	1a	Semi-IPN polyacrylamide/chitosan (PAAm/chitosan)	As(V)	17.8 mg g−1 (Abstract); 20 mg g−1 at 1000 mg L−1 (Conclusions)	experimental (no isotherm model fitted)	5.0 (optimum; pH study 2–11, Fig. 10A)	n.r.	10–1000 mg L−1 As(V) (§3.4.5, Fig. 13A)	0.05 g xerogel; 10 mL (Conclusions) → 5 g L−1	n.r.	n.r.	dry xerogel powder (§2.2)	Abstract; §2.2; §3.4.2 Fig. 10A; §3.4.5 Fig. 13A; §4	Two values (17.8 vs 20 mg g−1). Single-polymer comparator.

H. Zhang et al., ref. 67 (10.1016/j.ijbiomac.2019.05.185)	1a	Magnetic bentonite/carboxymethyl chitosan/sodium alginate beads	Cu^{2+}	56.79 mg g−1 (Langmuir q_max at 303.2 K, Table 3)	model-derived (Langmuir)	5.0 (isotherm, §3.12.2); optimum from pH 2.0–6.3 series	Langmuir (R2 higher than Freundlich, Table 3)	ambiguous: §3.12.2 states 25 mL of 50 mg L−1; §2.4 gives 5–150 mg L−1 for the experiments	50 mg in 25 mL (2 g L−1)	30 °C (303.2 K)	n.r.	dry beads (baked at 60 °C to constant weight, §2.3)	p. 2 §2.3–2.4; p. 8 §3.12.2; p. 9 Table 3	Table 1a pH corrected from n.s. to 5.0.
S. Chkirida et al., ref. 68 (10.1016/j.ijbiomac.2024.129389)	1c	Magnetic alginate-bentonite/chitosan core@shell beads (mAB@Cs)	Pb^{2+}, Cd^{2+}, Cu^{2+}, Zn^{2+}, Ni^{2+}, Hg^{2+}, Co^{2+} (each tested separately)	12–20 mg g−1, range across seven metals (Pb, Cd, Cu, Zn, Ni, Hg, Co), each tested separately; component values only in Fig. 6	experimental, single concentration	n.r. ('already optimised conditions' of the ciprofloxacin study)	none fitted for metals	50 mg L−1 (each metal)	0.5 g L−1; 120 min	ambient	n.r.	n.r.	p. 4 §2.6.2; p. 10 §3.4, Fig. 6	Correction: the 12–20 mg g−1 range comes from single-metal tests of seven metals, not from a Cu/Cd/Pb mixture; a separate competitive seven-metal test is shown only graphically. The real urban wastewater test (§2.6.3, §3.5) measured COD, BOD, N, P, K and bacteria, not metals.
Z. Zhang et al., ref. 70 (10.1016/j.ijbiomac.2025.144188)	1a	Sodium alginate/carboxylated-chitosan/attapulgite aerogel-PEI (ATP/CCS/SA-PEI)	Cu^{2+}	228.24 mg g−1 (Abstract, §3.5); Langmuir q_m 302.89 mg g−1 (Table 2)	ambiguous: 228.24 is called the maximum capacity from kinetics (§3.5) and 'theoretical maximum' in §4	4 (Cu; pH 1–6 studied)	Langmuir best fit (R2 0.9906 vs Freundlich 0.9831, D–R 0.9024)	isotherm range n.r. in the main text (300 mg L−1 in pH/dose tests)	ambiguous: '0.05 g L−1' stated with 50 mL, whereas 50 mg per 50 mL (1 g L−1) is used in §2.3.1	25 °C (pH/dose tests); 35 °C cited as optimum (§4)	n.r. (interfering ions tested separately)	lyophilised aerogel	p. 3 §2.3.1–2.3.2; p. 7 Table 2; p. 9 §3.5; p. 11 §4	Table 1a model corrected from n.s. to Langmuir; pH 4 added.
Z. Lin et al., ref. 71 (10.3390/polym13111891)	1a	Chitosan/calcium alginate/bentonite (CTS/CA/BT) hydrogel	Pb^{2+}	434.89 mg g−1	reported as 'maximum adsorption capacity' (Abstract); whether experimental or fitted q_m not stated in main text — non déterminable without Table S3	5.0 ± 0.1, initial, adjusted with HNO3/NaOH (§2.4 baseline)	Redlich–Peterson preferred (lower χ2/RMSE; §3.5, Fig. 10, Tables S3–S4); Langmuir, Freundlich, D–R also fitted	100–2500 mg L−1 Pb2+ (§2.5.2)	10 mg freeze-dried hydrogel; batch volume n.r. → g L−1 non déterminable (§2.4)	298, 308 and 318 K (isotherms, §2.5.2); baseline 25 ± 1 °C	n.r. for isotherms (KNO3/Mg(NO3)2 0.001–0.1 mol L−1 only in separate interference test, §3.3.4)	dry (freeze-dried) hydrogel mass (§2.4: 'm (mg) is the mass of the freeze-dried hydrogel')	Abstract; §2.4; §2.5.2; §3.3.2 Fig. 5; §3.5 Fig. 10	Table 1a pairs these 'maximum' values with Redlich–Peterson, which has no saturation parameter: the value is not an R–P parameter; correct the model column to

														'max. capacity (definition n.s.); R–P best fit'. Authors state precipitation at pH ≥ 6 (§3.3.2).
Z. Lin et al., ref. 71 (10.3390/polym13111891)	1a	Chitosan/calcium alginate/bentonite (CTS/CA/BT) hydrogel	Cu^{2+}	115.30 mg g−1	reported as 'maximum adsorption capacity' (Abstract); whether experimental or fitted q_m not stated in main text — non déterminable without Table S3	5.0 ± 0.1, initial, adjusted with HNO3/NaOH (§2.4 baseline)	Redlich–Peterson preferred (lower χ2/RMSE; §3.5, Fig. 10, Tables S3–S4); Langmuir, Freundlich, D–R also fitted	25–500 mg L−1 Cu2+ (§2.5.2)	10 mg freeze-dried hydrogel; batch volume n.r. → g L−1 non déterminable (§2.4)	298, 308 and 318 K (isotherms, §2.5.2); baseline 25 ± 1 °C	n.r. for isotherms (KNO3/Mg(NO3)2 0.001–0.1 mol L−1 only in separate interference test, §3.3.4)	dry (freeze-dried) hydrogel mass (§2.4: 'm (mg) is the mass of the freeze-dried hydrogel')	Abstract; §2.4; §2.5.2; §3.3.2 Fig. 5; §3.5 Fig. 10	Table 1a pairs these 'maximum' values with Redlich–Peterson, which has no saturation parameter: the value is not an R–P parameter; correct the model column to 'max. capacity (definition n.s.); R–P best fit'. Authors state precipitation at pH ≥ 6 (§3.3.2).
Z. Lin et al., ref. 71 (10.3390/polym13111891)	1a	Chitosan/calcium alginate/bentonite (CTS/CA/BT) hydrogel	Cd^{2+}	102.38 mg g−1	reported as 'maximum adsorption capacity' (Abstract); whether experimental or fitted q_m not stated in main text — non déterminable without Table S3	5.0 ± 0.1, initial, adjusted with HNO3/NaOH (§2.4 baseline)	Redlich–Peterson preferred (lower χ2/RMSE; §3.5, Fig. 10, Tables S3–S4); Langmuir, Freundlich, D–R also fitted	25–500 mg L−1 Cd2+ (§2.5.2)	10 mg freeze-dried hydrogel; batch volume n.r. → g L−1 non déterminable (§2.4)	298, 308 and 318 K (isotherms, §2.5.2); baseline 25 ± 1 °C	n.r. for isotherms (KNO3/Mg(NO3)2 0.001–0.1 mol L−1 only in separate interference test, §3.3.4)	dry (freeze-dried) hydrogel mass (§2.4: 'm (mg) is the mass of the freeze-dried hydrogel')	Abstract; §2.4; §2.5.2; §3.3.2 Fig. 5; §3.5 Fig. 10	Table 1a pairs these 'maximum' values with Redlich–Peterson, which has no saturation parameter: the value is not an R–P parameter; correct the model column to 'max. capacity (definition n.s.); R–P best fit'. Authors state precipitation at pH ≥ 6 (§3.3.2).
M. Kuczajowska-Zadrożna et al., ref. 72 (10.1016/j.jece.2020.103878)	1a	Chitosan immobilized in alginate beads (ALG-CHz)	Cu^{2+}	527.31 mg g−1 (Sips q_max, Table 2)	Sips q_max (model-derived); Langmuir q_max 121.0 (Cu) / 113.92 (Cd) mg g−1	5.0 (isotherms, Fig. 4 caption); pH 2–9 studied,	Freundlich, Langmuir, Sips, Langmuir 2	1–600 mg L−1	4.5 g dry mass L−1 (50 mL)	25 °C (Fig. 4); 'room temperature' in §2.2; 120 min	n.r.	dry mass (mg g−1 d.m.)	p. 2 §2.2; p. 5 Fig. 4; p. 6 Table 2	Calculated bound: C0,max/dose = 600/4.5 = 133 mg g−1; the

						adjusted with 1 M HNO_3/NaOH	fitted; Sips best (lowest ARE, Fig. 5)							Sips q_max is an extrapolation about four times above any attainable uptake. Table 1a pH corrected from 'pH-independent' to 5.0.
M. Kuczajowska-Zadrożna et al., ref. 72 (10.1016/j.jece.2020.103878)	1a	Chitosan immobilized in alginate beads (ALG-CHz)	Cd^{2+}	207.02 mg g−1 (Sips q_max, Table 2)	Sips q_max (model-derived); Langmuir q_max 121.0 (Cu) / 113.92 (Cd) mg g−1	5.0 (isotherms, Fig. 4 caption); pH 2–9 studied, adjusted with 1 M HNO_3/NaOH	Freundlich, Langmuir, Sips, Langmuir 2 fitted; Sips best (lowest ARE, Fig. 5)	1–600 mg L−1	4.5 g dry mass L−1 (50 mL)	25 °C (Fig. 4); 'room temperature' in §2.2; 120 min	n.r.	dry mass (mg g−1 d.m.)	p. 2 §2.2; p. 5 Fig. 4; p. 6 Table 2	Calculated bound 133 mg g−1 (as above); Sips q_max exceeds it. pH corrected to 5.0.
M. F. Hamza et al., ref. 73 (10.3390/ma14092189)	1a	Alginate-chitosan biopolymer composite (1:3 ratio)	Cd^{2+}	0.81 mmol g−1 (≈91.1 mg g−1; pH-study uptake, Abstract/§3.2.1: 0.822 mmol g−1, 92.43 mg g−1); isotherm exp. max 1.894 mmol g−1 (Table 2)	experimental (pH study); NOT the isotherm maximum	pH0 5.0 (isotherms, §3.2.3); pHeq ≈ 4 (§3.2.1); pH not fixed during sorption, final pH recorded (§2.4)	Langmuir and Sips fitted (Table 2); Langmuir closer for Cd, Sips for Pb (§3.2.3)	10–500 mg L−1 (isotherms, §2.4; Fig. 5 caption)	1 g L−1 (isotherms; §2.4)	22 ± 2 °C (§2.4); 23 ± 3 °C (Fig. 3, 5 captions)	n.r. for sorption tests (0.1 M NaCl only for pHPZC, §2.3)	dry sorbent (dried at 50 °C for 10 h, §2.2)	Abstract; §2.2–2.4; §3.2.1 Fig. 3; §3.2.3 Fig. 5; Tables 2–3	ERROR in Table 1a: 0.81 mmol Cd g−1 and 0.41 mmol Pb g−1 (Abstract) are uptakes from the pH study (C0 ≈ 0.96 mmol Cd L−1 / 0.48–0.51 mmol Pb L−1, SD 1 g L−1, Fig. 3), not isotherm capacities; the 'Langmuir / Sips' label does not apply to them. Isotherm values for CA#3 (Table 2/3): Cd exp. qm 1.894 mmol g−1 (≈212.9 mg g−1), Langmuir qm,L 1.926; Pb exp. qm 1.054 mmol g−1 (≈218.4 mg g−1), Langmuir 1.248, Sips

														1.118 mmol g−1.
M. F. Hamza et al., ref. 73 (10.3390/ma14092189)	1a	Alginate-chitosan biopolymer composite (1:3 ratio)	Pb^{2+}	0.41 mmol g−1 (≈85.0 mg g−1; pH study; §3.2.1: 0.42 mmol g−1, 87.02 mg g−1); isotherm exp. max 1.054 mmol g−1 (Table 2)	experimental (pH study); NOT the isotherm maximum	pH0 5.0 (isotherms, §3.2.3); pHeq ≈ 4 (§3.2.1); pH not fixed during sorption, final pH recorded (§2.4)	Langmuir and Sips fitted (Table 2); Langmuir closer for Cd, Sips for Pb (§3.2.3)	10–500 mg L−1 (isotherms, §2.4; Fig. 5 caption)	1 g L−1 (isotherms; §2.4)	22 ± 2 °C (§2.4); 23 ± 3 °C (Fig. 3, 5 captions)	n.r. for sorption tests (0.1 M NaCl only for pHPZC, §2.3)	dry sorbent (dried at 50 °C for 10 h, §2.2)	Abstract; §2.2–2.4; §3.2.1 Fig. 3; §3.2.3 Fig. 5; Tables 2–3	ERROR in Table 1a: 0.81 mmol Cd g−1 and 0.41 mmol Pb g−1 (Abstract) are uptakes from the pH study (C0 ≈ 0.96 mmol Cd L−1 / 0.48–0.51 mmol Pb L−1, SD 1 g L−1, Fig. 3), not isotherm capacities; the 'Langmuir / Sips' label does not apply to them. Isotherm values for CA#3 (Table 2/3): Cd exp. qm 1.894 mmol g−1 (≈212.9 mg g−1), Langmuir qm,L 1.926; Pb exp. qm 1.054 mmol g−1 (≈218.4 mg g−1), Langmuir 1.248, Sips 1.118 mmol g−1.
X. Liu et al., ref. 74 (10.1039/D5RA00940E)	1a	EDTA-chitosan/alginate porous composite beads (EC-AB)	Pb^{2+}	qm,exp 504.11 mg g−1 (experimental); Langmuir qm,cal 492.97 mg g−1 (Table 3)	experimental maximum (the Langmuir value is 492.97)	6 (isotherms, §2.5); pH study 1–6, removal high over the range (Fig. 4A)	Langmuir preferred (lower RMSE); Freundlich also fitted (Table 3)	15–1000 mg L−1 Pb(II) (isotherms, §2.5)	10 mg adsorbent in 10 mL → 1 g L−1 (§2.5)	25 °C (298 K) (§2.5; Abstract)	n.r. for isotherms; Na+/Mg2+/Ca2+/Cl−/NO3−/SO42− 0–100 mmol L−1 in separate interference tests (§2.5, §3.2.5)	dry weight stated for the column packing (0.5 g, §2.6); batch mass basis not stated explicitly — non déterminable	Abstract; §2.5; §3.2.2 Fig. 4; §3.2.4 Fig. 6, Table 3	CORRECTIONS for Table 1a: pH column 'wide pH range' → 'pH 6 (isotherm)'; model column: 504.11 is the experimental maximum, Langmuir qm = 492.97 mg g−1.
E. H. Ablouh et al., ref. 75 (10.1186/s42834-019-	1a	Chitosan microspheres/sodium alginate hybrid beads (CSM/SA)	Pb^{2+}	Langmuir qmax 189	Table 1a value (180) matches	5.2 initial (optimu	Langmuir best fit	25–300 mg L−1 (Batch	0.06 g (isotherm	25 °C (Fig. 6e caption)	n.r. (0.01 M NaCl only for PZC)	dried beads	Abstract; Batch	CORRECTION: Table 1a

0004-9)				mg g−1; qe,exp 161 mg g−1 (Table 2); Abstract gives 180, Conclusions 188 mg g−1	neither Table 2 qmax (189) nor qe,exp (161)	m; Fig. 6e)	(R2 0.992 Pb, 0.979 Cr); Freundlich also fitted (Table 2)	experiments; Fig. 6e)	series, Fig. 6e caption); solution volume n.r. → g L−1 non déterminable			('washed … and then dried', Preparation section); drying conditions n.r.	experiments section; Fig. 6; Adsorption isotherms section; Table 2; Conclusions	'180 mg g−1' is the Abstract figure; Table 2 gives Langmuir qmax 189 and experimental 161 mg g−1. Pb(OH)2 precipitation observed above pH 6.
E. H. Ablouh et al., ref. 75 (10.1186/s42834-019-0004-9)	1a	Chitosan microspheres/sodium alginate hybrid beads (CSM/SA)	Cr(VI)	Langmuir qmax 16 mg g−1; qe,exp 14 mg g−1 (Table 2)	model-derived (Langmuir); experimental 14 mg g−1	3.0 initial (optimum; Fig. 6e)	Langmuir best fit (R2 0.992 Pb, 0.979 Cr); Freundlich also fitted (Table 2)	25–300 mg L−1 (Batch experiments; Fig. 6e)	0.06 g (isotherm series, Fig. 6e caption); solution volume n.r. → g L−1 non déterminable	25 °C (Fig. 6e caption)	n.r. (0.01 M NaCl only for PZC)	dried beads ('washed … and then dried', Preparation section); drying conditions n.r.	Abstract; Batch experiments section; Fig. 6; Adsorption isotherms section; Table 2; Conclusions	Table 1a value 16 = Langmuir qmax.
H.-C. Tao et al., ref. 77 (10.1007/s10653-018-0137-5)	1a	Magnetic chitosan/sodium alginate gel bead (MCSB, Fe3O4)	Cu^{2+}	124.53 mg g−1 (Langmuir, 'theoretical maximum'); experimental maximum 90.88 ± 0.8 mg g−1 at 60–80 mg L−1	model-derived (Langmuir)	5.0	Langmuir preferred to Freundlich	10–80 mg L−1	12 mg in 50 mL (0.24 g L−1)	25 °C	n.r.	ambiguous: beads stored in water before use; whether the 12 mg refers to wet or dry mass is not stated	p. 3 (batch adsorption experiments); p. 6 (effect of initial concentration); p. 9 (isotherms)	
C. Gao et al., ref. 78 (10.1016/j.carbpol.2020.117564)	1a	Alginate/melamine/chitosan aerogel	Pb^{2+}	1331.6 mg g−1 (Langmuir q_m, non-linear fit, Table 2)	model-derived (Langmuir)	5.5 (optimum; pH 1–6 studied)	Langmuir (R2 0.9809) preferred to Freundlich (0.9480)	100–2000 mg L−1	20 mg in 20 mL (1 g L−1)	room temperature (n.r. as a value)	n.r. (binary competition tests separately, 500 mg L−1)	freeze-dried aerogel	p. 3 §2.4; p. 6 Table 2; p. 7 §3.6, Fig. 5b	Regeneration: capacity about 60 % of the initial value after eight adsorption–desorption cycles (0.05 M HNO3), i.e. a capacity-retention value, not a desorption efficiency.
W. Zhang et al., ref. 79 (10.1016/j.jclepro.2019.05.219)	1a	Multicavity TETA-chitosan/alginate composite beads (HCTA)	Cr(VI)	≈291.3 mg g−1 (experimental maximum, HCTA-2 beads)	experimental	3.0 (isotherms, Fig. 2 caption); initial pH 2–11 studied	Freundlich preferred (adj. R2 0.9593–0.9950) to Langmui	20–1000 mg L−1	1.0 g L−1 (0.05 g in 50 mL); 1440 min	20 °C	n.r. for isotherms (salts 0–0.1 M tested separately, §3.2)	dried adsorbent (§2.4)	p. 3 §2.4; p. 5 §3.1.2, Fig. 2	Table 1a model corrected from n.s. to Freundlich best fit. Cr(III) was measured, so part of

							r (0.8280 – 0.9448), non-linear fits (Table 1)							the removal involves reduction. A column study is also reported (§2.5, §3.1.4).
L. S. Alqarni et al., ref. 80 (10.1016/j.ijbiomac.2024.129989)	1a	Chitosan-alginate@Fe/Mn mixed oxide nanocomposite (Cs.Alg@Fe/Mn)	Cr(VI)	455.93 mg g−1 (Langmuir Q_max, Table 4)	model-derived (Langmuir)	4 (optimum; pH 1–7 studied)	Langmuir (R2 0.989 in Table 4; text states 0.999), Freundlich 0.975, Temkin 0.938	25–1000 mg L−1	1 g L−1 (optimum, §3.2.4); 50 mL	25 °C (298–323 K studied)	n.r. (co-ions tested separately, §3.8)	n.r.	p. 3 §2.3; p. 6 §3.2.4; p. 11 Table 4	Table 1a capacity corrected from n.s. to 455.93 mg g−1 and pH from n.s. to 4.
S. Gokila et al., ref. 81 (10.1016/j.ijbiomac.2017.05.117)	1a	Chitosan-alginate nanocomposite	Cr(VI)	39.59 mg g−1 (Langmuir C_max, linearised fit, Table 2)	model-derived (Langmuir, linear)	5 (optimum; pH 2–8 studied)	Freundlich R2 0.970 > Langmuir 0.943 (Table 2)	100 mg L−1 in the parameter series; isotherm range n.r.	n.r. for isotherms	ambient	n.r.	n.r.	p. 2 §2 (adsorption procedure); p. 4–5 §3.6.1; p. 8 Table 2	Table 1a capacity corrected from n.s. to 39.59 mg g−1 (Langmuir), model from n.s. to 'Freundlich best fit'.
R. Dubey et al., ref. 82 (10.1016/j.enmm.2016.06.008)	1a	Chitosan-alginate nanoparticles (CANPs)	Hg^{2+}	217.39 mg g−1 (Langmuir Q0, Table 1)	model-derived (Langmuir)	5	Langmuir R2 0.961 > Freundlich 0.592, Temkin 0.421, D–R 0.695	4–12 mg L−1 (§3.6.4)	0.1 g in 10 mL (10 g L−1); 90 min	30 ± 0.2 °C	n.r.	n.r.	p. 2 §2.5; p. 8 §3.6.4; p. 9 Fig. 10; p. 10 Table 1	Calculated bound: at most (12 mg L−1 × 0.010 L)/0.1 g = 1.2 mg g−1 (≈0.8 mg g−1 at the 64 % removal reported), so Q0 is an extrapolation more than 100 times above the measured range.
W. Li et al., ref. 83 (10.1016/j.ijbiomac.2021.07.181)	1a	Mesoporous nanocellulose/sodium alginate/carboxymethyl-chitosan gel beads	Cu^{2+}	169.94 mg g−1 (Langmuir)	model-derived (Langmuir Q_max, Table 3); experimental maxima 149.83 (Cu) and 456.42 (Pb) mg g−1 (§4.4.4)	n.r. for isotherms (pH 2.0–5.0 studied; capacity highest at 5.0)	Langmuir (R2 0.9927 Cu, 0.9940 Pb); Freundlich also fitted	50–300 mg L−1	30 mg in 50 mL (0.6 g L−1); 24 h	30 °C	n.r. (background ions tested separately)	freeze-dried aerogel beads	p. 3 §3.1; p. 6 §4.4.4, Table 3	
W. Li et al., ref. 83 (10.1016/j.ijbiomac.2021.07.181)	1a	Mesoporous nanocellulose/sodium alginate/carboxymethyl-chitosan gel beads	Pb^{2+}	472.59 mg g−1 (Langmuir)	model-derived (Langmuir Q_max, Table 3); experimental maxima 149.83 (Cu) and 456.42	n.r. for isotherms (pH 2.0–5.0 studied; capacity	Langmuir (R2 0.9927 Cu, 0.9940 Pb);	50–300 mg L−1	30 mg in 50 mL (0.6 g L−1); 24 h	30 °C	n.r. (background ions tested separately)	freeze-dried aerogel beads	p. 3 §3.1; p. 6 §4.4.4, Table 3	

					(Pb) mg g−1 (§4.4.4)	highest at 5.0)	Freundlich also fitted							
Y. Huang et al., ref. 84 (10.1016/j.cej.2018.01.071)	1a	DTPA-chitosan/alginate composite beads (Ca-DTCS/ALG)	Cu^{2+}	107.5 mg g−1 (q_e,exp, Ca-DTCS/ALG beads, Table 2); Langmuir 103.3, Sips 110.5 mg g−1	experimental	3.0	Langmuir, Freundlich, Sips (non-linear); Sips lowest RMSE	20–500 mg L−1	1 g L−1 dry-weight equivalent; 36 h	28 °C	n.r. (Ca, Na, Al salts tested separately)	dry-weight equivalent of gel beads	p. 3 §2.5; p. 7 Fig. 5, Table 2	Correction: the lower value 106.0 mg g−1 in Table 1a belongs to the DTCS powder, not to the composite beads; the composite value is 107.5 mg g−1.
R. Ghubayra et al., ref. 85 (10.1016/j.ijbiomac.2024.133513)	1a	TiO2@chitosan/alginate nanocomposite sponge	As(V)	202.27 mg g−1 (Box–Behnken response-surface estimate); concentration series 45.19–202 mg g−1 (§3.2.3)	model-derived (response-surface prediction)	5.0	Langmuir, Freundlich, D–R, Temkin, Jossens, Toth fitted; parameters in the article's Table S2 (not available)	50–440 mg L−1	10 mg in 25 mL (0.4 g L−1); 90 min	25 °C	n.r. (coexisting ions tested separately)	n.r.	p. 3 §2.3; p. 9 §3.2.3–3.3; p. 17 §3.13 (RSM optimum)	Isotherm parameters n.v. (supplementary file not available).
A. G. Contreras-Cortés et al., ref. 86 (10.3390/polym11020222)	1a	Chitosan-alginate + Aspergillus australensis biomass beads	Cu^{2+}	26.1 mg g−1 (experimental, highest q at C0 176.0 mg L−1, Table 2); Langmuir qmax 26.56 mg g−1 (b = 0.358, R2 = 0.927; §3.3, Fig. 5)	range combines experimental maximum (26.1) and Langmuir qmax (26.56)	5.0 (isotherm series 50–200 ppm, §2.6.2); 4.5–5.5 tested at 20 ppm	Langmuir (only model fitted, §2.8)	20, 50, 100 and 200 ppm Cu2+ nominal; measured 20.7–176.0 mg L−1 (Table 2)	0.15 g lyophilized beads in 150 mL → 1 g L−1 (§2.6.2)	35 °C (§2.6.1–2.6.2)	n.r.	lyophilized (dry) composite beads; Eq. 1 defines m as 'dried biomass (g)' (§2.7)	Abstract; §2.6.1–2.8; §3.3, Table 2, Fig. 5	Table 1a '26.1–26.6' is correct but mixes an experimental value and a fitted qmax (26.56 → round to 26.6); specify. Authors avoided pH > 6 to prevent Cu(OH)2 precipitation (§2.6.1, §3.3).
E. Matei et al., ref. 87 (10.37358/MP.21.1.5441)	1b	Alginate/chitosan/glass bubbles adsorbent	Cu^{2+}	n.r. as a capacity; % removal only: ~80 % at 20 min (ratio 1:50) and max. 87 % at 125 min (ratio 1:100) (PDF p4, Fig. 3; p7 Conclusions	percentage removal (no capacity value tabulated in text)	n.r.	n.r. (no isotherm model fitted; Table 1a 'Langmuir' is not supported)	4 mg L−1 Cu(II) for the tests (PDF p3); standards 1–6 mg L−1 (PDF p2)	10 g or 5 g microspheres in 500 mL → 20 or 10 g L−1 (PDF p3)	25 °C (PDF p3)	n.r. (Cu(NO3)2 stock in 0.5 M HNO3, dilution not specified)	dried microspheres (30 °C, 48 h; PDF p2)	PDF p2 (preparation), p3 (testing), p4 (Fig. 3, regeneration), p7 (Conclusions)	CORRECTION: entry belongs to Table 1b (percentage removal), not 1a; 'Langmuir' to be removed; synthetic Cu(NO3)2 solution,

				)										not a real wastewater; 5 regeneration cycles tested (Fig. 4).
J. F. de Souza et al., ref. 88 (10.1007/s10924-022-02599-8)	1a	Alginate/phosphine-functionalized chitosan beads (Alg/CSPPh2)	Cd^{2+}	78.2 mg g−1 (experimental)	experimental (highest q_t at 150 mg L−1 in the kinetic series)	6.0 (selected; initial pH 7.0 in screening)	Freundlich best fit (R2 0.980 Cd, 0.989 Al); Langmuir q_max 73.3 (Cd) and 346.3 (Al) mg g−1; Temkin also fitted	10–150 mg L−1	50 mg in 50 mL (1 g L−1); 3 h	25 °C	n.r.	oven-dried beads (45 °C, 48 h)	p. 2 (bead preparation); p. 3 (batch experiments); p. 8–9 (kinetics, isotherms, Table 2)	The Langmuir q_max for Al3+ (346.3 mg g−1) exceeds the attainable 150 mg g−1 (C0,max V/m), but Table 1a uses the experimental value. Al3+ at pH 6 lies near the Al(OH)3 solubility minimum (Section 5.3).
J. F. de Souza et al., ref. 88 (10.1007/s10924-022-02599-8)	1a	Alginate/phosphine-functionalized chitosan beads (Alg/CSPPh2)	Al3+	81.5 mg g−1 (experimental)	experimental (highest q_t at 150 mg L−1 in the kinetic series)	6.0 (selected; initial pH 7.0 in screening)	Freundlich best fit (R2 0.980 Cd, 0.989 Al); Langmuir q_max 73.3 (Cd) and 346.3 (Al) mg g−1; Temkin also fitted	10–150 mg L−1	50 mg in 50 mL (1 g L−1); 3 h	25 °C	n.r.	oven-dried beads (45 °C, 48 h)	p. 2 (bead preparation); p. 3 (batch experiments); p. 8–9 (kinetics, isotherms, Table 2)	The Langmuir q_max for Al3+ (346.3 mg g−1) exceeds the attainable 150 mg g−1 (C0,max V/m), but Table 1a uses the experimental value. Al3+ at pH 6 lies near the Al(OH)3 solubility minimum (Section 5.3).
S. Tang et al., ref. 89 (10.1016/j.cej.2020.124728)	1a	Chitosan/sodium alginate/Ca2+ double-network hydrogel (CTS/SA/Ca2+ PCDNH)	Pb^{2+}	176.50 mg g−1	experimental, single concentration (composition screening)	n.r. ('adjusted to the desired value')	Freundlich higher R2 at 298 and 308 K, Langmuir at 318 K; Langmuir–Freundlich and D–R also fitted (Table 3)	200 mg L−1 (capacity test)	25 mg in 25 mL (1 g L−1); 8 h	298 K	n.r.	freeze-dried hydrogel	p. 3 §2.4.1; p. 7 §3.5; p. 9 Table 3	
S. Tang et al., ref. 89 (10.1016/j.cej.2020.1247	1a	Chitosan/sodium alginate/Ca2+ double-network hydrogel	Cu^{2+}	70.83 mg g−1	experimental, single	n.r. ('adjuste	Freundlich	200 mg L−1 (capacity	25 mg in 25 mL (1	298 K	n.r.	freeze-dried	p. 3 §2.4.1; p. 7 §3.5;	

28)		(CTS/SA/Ca2+ PCDNH)			concentration (composition screening)	d to the desired value')	higher R2 at 298 and 308 K, Langmuir at 318 K; Langmuir–Freundlich and D–R also fitted (Table 3)	test)	g L−1); 8 h			hydrogel	p. 9 Table 3	
S. Tang et al., ref. 89 (10.1016/j.cej.2020.124728)	1a	Chitosan/sodium alginate/Ca2+ double-network hydrogel (CTS/SA/Ca2+ PCDNH)	Cd^{2+}	81.25 mg g−1	experimental, single concentration (composition screening)	n.r. ('adjusted to the desired value')	Freundlich higher R2 at 298 and 308 K, Langmuir at 318 K; Langmuir–Freundlich and D–R also fitted (Table 3)	200 mg L−1 (capacity test)	25 mg in 25 mL (1 g L−1); 8 h	298 K	n.r.	freeze-dried hydrogel	p. 3 §2.4.1; p. 7 §3.5; p. 9 Table 3	
I. Jioui et al., ref. 90 (10.1007/s11356-023-29890-y)	1a	Alginate-chitosan/hydroxyapatite from rock phosphate (Alg-Cs/HAP)	Cu^{2+}	208.34 mg g−1 (Langmuir q_m, Table 1); experimental maximum 197.5 mg g−1 at 250 mg L−1	model-derived (Langmuir)	5 (Cu; pH 3–5.5 studied; precipitation observed above 5.5)	Langmuir (R2 0.994); Freundlich and Temkin also fitted	25–250 mg L−1	27 mg in 25 mL (≈1.1 g L−1); 120 min	room temperature (25 °C in batch description)	n.r.	ambiguous: dose series given as wet mass (0.1–1 g) and also as dried mass	p. 4 (batch adsorption); p. 7 (pH, dose); p. 9 Fig. 12; p. 13 Table 1	Table 1a pH corrected from n.s. to 5 and model from n.s. to Langmuir.
R. Sasirekha et al., ref. 91 (10.1016/j.mtcomm.2023.106339)	1a	Chitosan nanoparticles/alginate/CMC/hydroxyapatite biocomposite	Cu^{2+}	512.79 mg g−1 (Langmuir C_max, linearised fit, Table 1)	model-derived (Langmuir, linear)	6	Freundlich R2 0.9984 > Langmuir 0.9357	50–500 mg L−1	0.1 g in 50 mL (2 g L−1); 60 min	28 °C	n.r.	n.r.	p. 2 §2.5; p. 7 Table 1; p. 8 §3.9	Calculated bound: C0,max V/m = 500 × 0.050/0.1 = 250 mg g−1; the Langmuir C_max is about twice the attainable uptake. Table 1a capacity corrected from n.s.
X. Yi et al., ref. 92 (10.1007/s11356-017-0802-8)	1a	Chitosan/calcium alginate/Fe3O4 hydrogel microsphere (CAF)	Cu^{2+}	145.39 mg g−1 (CAF)	model-derived (Langmuir q_m, Table 2)	4.5–5	Langmuir best fit (R2 0.975 CAF, 0.994 NACAF); Freundli	20–800 mg L−1	0.5 g L−1 (5 mg in 10 mL); 4 h	25 °C	n.r.	freeze-dried microspheres	p. 2 (preparation); p. 3 (batch adsorption test); p. 9 Fig. 8, Table 2	Table 1a pH corrected from n.s. to 4.5–5 and model from n.s. to Langmuir.

							ch and Temkin also fitted							
X. Yi et al., ref. 92 (10.1007/s11356-017-0802-8)	1a	NaOH-modified chitosan/calcium alginate/Fe_3O_4 (NACAF)	Cu^{2+}	261.31 mg g−1 (NACAF)	model-derived (Langmuir q_m, Table 2)	4.5–5	Langmuir best fit (R2 0.975 CAF, 0.994 NACAF); Freundlich and Temkin also fitted	20–800 mg L−1	0.5 g L−1 (5 mg in 10 mL); 4 h	25 °C	n.r.	freeze-dried microspheres	p. 2 (preparation); p. 3 (batch adsorption test); p. 9 Fig. 8, Table 2	Table 1a pH corrected from n.s. to 4.5–5 and model from n.s. to Langmuir.
K. Vijayalakshmi et al., ref. 93 (10.1016/j.ijbiomac.2017.04.120)	1a	Nanochitosan/sodium alginate/microcrystalline cellulose beads	Pb^{2+}	114.47 mg g−1 (Langmuir C_max, Table 2); D–R q_m 39.33 mg g−1	model-derived	6 (pH 4–6 studied)	Freundlich best fit (R2 0.9962; Langmuir 0.9613)	62.5–1000 mg L−1	1 g in 100 mL (10 g L−1); 1 h	30 °C (optimum 50 °C in Abstract)	n.r.	n.r. (beads used after rinsing)	p. 3 §4.4; p. 6 §5.5.4; p. 8 Table 2; p. 9 Table 4	Correction: the value 318.47 mg g−1 in Table 1a does not appear in the article; replaced by the Langmuir C_max 114.47 mg g−1.
H. Yue et al., ref. 94 (10.1016/j.seppur.2021.119767)	1a	EDTA-modified chitooligosaccharide/sodium alginate/Ca^{2+} hydrogel (ECSDNH)	Zn^{2+}	160.19 mg g−1	model-derived (Langmuir Q_m at 298 K, Table 2)	n.r. for isotherms (pH 2–7 studied; screening at pH 7)	Langmuir (fits 'fairly well'; Table 2)	n.r. in §2.3.3	25 mg in 25 mL (1 g L−1); 12 h	298 K (also 308 and 318 K)	n.r.	freeze-dried hydrogel	p. 3 §2.3.1, §2.3.3; p. 8 Table 2	Table 1a model corrected from n.s. to Langmuir. Screening at pH 7 approaches hydroxide saturation for Zn^{2+}.
H. Yue et al., ref. 94 (10.1016/j.seppur.2021.119767)	1a	EDTA-modified chitooligosaccharide/sodium alginate/Ca^{2+} hydrogel (ECSDNH)	Ni^{2+}	72.67 mg g−1	model-derived (Langmuir Q_m at 298 K, Table 2)	n.r. for isotherms (pH 2–7 studied; screening at pH 7)	Langmuir (fits 'fairly well'; Table 2)	n.r. in §2.3.3	25 mg in 25 mL (1 g L−1); 12 h	298 K (also 308 and 318 K)	n.r.	freeze-dried hydrogel	p. 3 §2.3.1, §2.3.3; p. 8 Table 2	Table 1a model corrected from n.s. to Langmuir. Screening at pH 7 approaches hydroxide saturation for Zn^{2+}.
H. Yue et al., ref. 94 (10.1016/j.seppur.2021.119767)	1a	EDTA-modified chitooligosaccharide/sodium alginate/Ca^{2+} hydrogel (ECSDNH)	Mn^{2+}	52.43 mg g−1	model-derived (Langmuir Q_m at 298 K, Table 2)	n.r. for isotherms (pH 2–7 studied; screening at pH 7)	Langmuir (fits 'fairly well'; Table 2)	n.r. in §2.3.3	25 mg in 25 mL (1 g L−1); 12 h	298 K (also 308 and 318 K)	n.r.	freeze-dried hydrogel	p. 3 §2.3.1, §2.3.3; p. 8 Table 2	Table 1a model corrected from n.s. to Langmuir. Screening at pH 7 approaches hydroxide saturation for Zn^{2+}.
H. Javadian et al., ref. 95 (10.1016/j.ijbiomac.2020.03.131)	1b	Calcium alginate/carboxymethyl chitosan microbiocomposite ($Ni_{0.2}Zn_{0.2}Fe_{2.6}O_4$)	Nd^{3+}	94.61 % (predicted 95.72 %)	experimental removal in the confirmation run; response-surface	5.5	Freundlich preferred to Langmui	30 mg L−1 of each ion, ternary 1:1:1	0.1 g in 50 mL (2 g L−1); 53 min	25 °C (25–45 °C in thermodynamic series)	n.r. for removal run (ionic-strength effect tested separately, §3.8)	dried powder (50 °C)	p. 2 §2.5; p. 8 Table 3 and §3.5; p. 9 §3.7; p. 13 §3.11	Column (§2.7, Table 7): 0.3 g sorbent + 1.8 g sand,

					predictions in brackets		r (isotherms 30–180 mg L−1, 0.09 g, pH 5.5)						(column), Table 7	i.d. 0.5 cm, bed 6.5 cm, 1 mL min−1, ternary 30 mg L−1 feed; breakthrough (Ct/C0 = 0.05) at about 95, 105 and 120 min and exhaustion at 410, 430 and 440 min (Nd, Tb, Dy); breakthrough-curve q_e 22.70, 22.70 and 25.54 mg g−1; column efficiency 46–49 %. Regeneration with 0.2 M HNO3: removal decreased by 2.54, 1.63 and 1.16 points after four cycles. Correction: Table 1b reported the predicted value.
H. Javadian et al., ref. 95 (10.1016/j.ijbiomac.2020.03.131)	1b	Calcium alginate/carboxymethyl chitosan microbiocomposite (Ni0.2Zn0.2Fe2.6O4)	Tb3+	96.73 % (predicted 96.17 %)	experimental removal in the confirmation run; response-surface predictions in brackets	5.5	Freundlich preferred to Langmuir (isotherms 30–180 mg L−1, 0.09 g, pH 5.5)	30 mg L−1 of each ion, ternary 1:1:1	0.1 g in 50 mL (2 g L−1); 53 min	25 °C (25–45 °C in thermodynamic series)	n.r. for removal run (ionic-strength effect tested separately, §3.8)	dried powder (50 °C)	p. 2 §2.5; p. 8 Table 3 and §3.5; p. 9 §3.7; p. 13 §3.11 (column), Table 7	Column (§2.7, Table 7): 0.3 g sorbent + 1.8 g sand, i.d. 0.5 cm, bed 6.5 cm, 1 mL min−1, ternary 30 mg L−1 feed; breakthrough (Ct/C0 = 0.05) at about 95, 105 and 120 min and exhaustion at 410, 430 and 440 min (Nd, Tb, Dy);

														breakthrough-curve q_e 22.70, 22.70 and 25.54 mg g−1; column efficiency 46–49 %. Regeneration with 0.2 M HNO3: removal decreased by 2.54, 1.63 and 1.16 points after four cycles.
H. Javadian et al., ref. 95 (10.1016/j.ijbiomac.2020.03.131)	1b	Calcium alginate/carboxymethyl chitosan microbiocomposite (Ni0.2Zn0.2Fe2.6O4)	Dy3+	98.83 % (predicted 99.44 %)	experimental removal in the confirmation run; response-surface predictions in brackets	5.5	Freundlich preferred to Langmuir (isotherms 30–180 mg L−1, 0.09 g, pH 5.5)	30 mg L−1 of each ion, ternary 1:1:1	0.1 g in 50 mL (2 g L−1); 53 min	25 °C (25–45 °C in thermodynamic series)	n.r. for removal run (ionic-strength effect tested separately, §3.8)	dried powder (50 °C)	p. 2 §2.5; p. 8 Table 3 and §3.5; p. 9 §3.7; p. 13 §3.11 (column), Table 7	Column (§2.7, Table 7): 0.3 g sorbent + 1.8 g sand, i.d. 0.5 cm, bed 6.5 cm, 1 mL min−1, ternary 30 mg L−1 feed; breakthrough (Ct/C0 = 0.05) at about 95, 105 and 120 min and exhaustion at 410, 430 and 440 min (Nd, Tb, Dy); breakthrough-curve q_e 22.70, 22.70 and 25.54 mg g−1; column efficiency 46–49 %. Regeneration with 0.2 M HNO3: removal decreased by 2.54, 1.63 and 1.16 points after four cycles.
J. D. Guerrero et al., ref. 96 (10.1016/j.ijbiomac.2023.126416)	1b	Chitosan/sodium alginate biocomposite (CSAR3-L, freeze-dried)	Cu^{2+}	94 % removal, q_e 186.6 mg g−1 (CSAR3-L, §3.1);	experimental removal; capacity also reported	6.0	Langmuir (R^2 0.998) > Freundlich (0.780)	10–500 mg L−1 (isotherms)	0.5 g L−1; 360 min	25 °C	n.r.	freeze-dried biocomposite	p. 3 §2.3; p. 7 §3.1; p. 14 §3.4, Fig. 13	Correction: only Cu(II) was studied (no Pb), and the material

				Langmuir q_{max} 300.3 mg g^{-1}, experimental 288.0 mg g^{-1} (CSAR3L-S40, §3.4)										codes in Table 1b were wrong (CSAR3-L).

Studies not verified in full text (n.v.; full text not accessible during this revision): refs 60 (Wiley), 69 and 76 (Springer), 5 entries in total. For these entries the conditions in Tables 1a–c are those recorded from the sources consulted.
